# Facilitating nitrogen accessibility to boron-rich covalent organic frameworks via electrochemical excitation for efficient nitrogen fixation

**DOI:** 10.1038/s41467-019-11846-x

**Published:** 2019-08-29

**Authors:** Sisi Liu, Mengfan Wang, Tao Qian, Haoqing Ji, Jie Liu, Chenglin Yan

**Affiliations:** College of Energy, Key Laboratory of Advanced Carbon Materials and Wearable Energy Technologies of Jiangsu Province, Soochow University, Suzhou, 215006 China

**Keywords:** Electrochemistry, Energy

## Abstract

Covalent organic frameworks with abundant active sites are potential metal-free catalysts for the nitrogen reduction reaction. However, the utilization ratio of active sites is restricted in an actual reaction process due to the limited nitrogen transport. Here, we demonstrate that facilitating the N_2_ accessibility to boron-rich covalent organic frameworks through electrochemical excitation can achieve highly efficient nitrogen reduction activity. Simulations show that the boron sites are bonded with nitrogenous species under electrochemical condition and the resultant amorphous phase of covalent organic frameworks has much stronger affinity toward N_2_ to enhance the molecule collision. Combined with experimental results, the excitation process is confirmed to be a virtuous cycle of more excited sites and stronger N_2_ affinity, which continuously proceed until the whole system reaches the optimum reaction status. As expected, the electrochemically excited catalyst delivers significantly enhanced reaction activity, with a high Faradaic efficiency of 45.43%.

## Abstract (web summary)

Covalent organic frameworks are potential catalysts for nitrogen reduction, but limited nitrogen transport restricts the utilization ratio of active sites. Here, the authors demonstrate that electrochemical excitation of covalent organic frameworks can lead to efficient nitrogen reduction.

## Introduction

The chemical ammonia (NH_3_) is essential for human beings and ecological system as a fertilizer feedstock and clean energy carrier^1–3^. Yet, industrial-scale ammonia production is monopolized by traditional Haber-Bosch process under drastic operating conditions, making the energy consumption and greenhouse gas emissions unavoidable all over the world^4–7^. While the main precursor N_2_ of this process is one of the most stable substances in nature, only low N_2_-to-NH_3_ conversion efficiency (~15%) can be achieved due to the extremely high bond energy of the N≡N covalent triple bond^8–10^. Theoretical studies and previous work have confirmed that N_2_ reduction to NH_3_ is feasible under ambient conditions when suitable voltage was applied^11,12^. Thus, direct electrocatalytic N_2_ fixation powered by renewable electricity is a very promising candidate as alternative of Haber-Bosch strategy for future use^13–15^.

Acting as the cornerstone of electrochemical catalysis, heterogeneous catalyst is crucial for the chemical transformation in nitrogen reduction reaction (NRR). The extended economic challenges especially encourage the development of metal-free catalysts with highly efficient active sites to increase the output and thus reduce the overall cost^16,17^. Specifically, boron sites doped in carbon framework induce electron deficiency that lead to significantly improved electrocatalytic activity, for instance, toward oxygen reduction reaction^18^ and CO_2_ reduction reaction^19^, and they thus can be extended to NRR^20–22^. However, the boron-doping amount in carbon is rather restricted so that the reaction activity is usually far from satisfaction^23^. Taking this into account, covalent organic frameworks (COFs), formed by repetitive organic molecules through strong covalent bonds, provide unlimited imagination for the design of catalysts with abundant active sites^24,25^. This class of material offers superiorities of modularity, porosity, stability and low density that endow them great potential in catalysis. In detail, regular extension of molecular units can create plentiful active sites in COFs, and desired structure and functions can be achieved by precisely controlling the building units^26,27^. Among various options, COFs linked by boronate ester (C_2_O_2_B) or boroxine (B_3_O_3_) rings contain large amount of boron atoms, featuring strong Lewis acidity that greatly promote the N_2_ (a weak Lewis base) adsorption^28^. Moreover, the chemical environment of boron site in COFs is tunable compared with traditional boron-doped carbon catalysts, which holds great potential to achieve further improvement in its intrinsic activity, and thus the overall NRR performance.

**Fig. 1:**
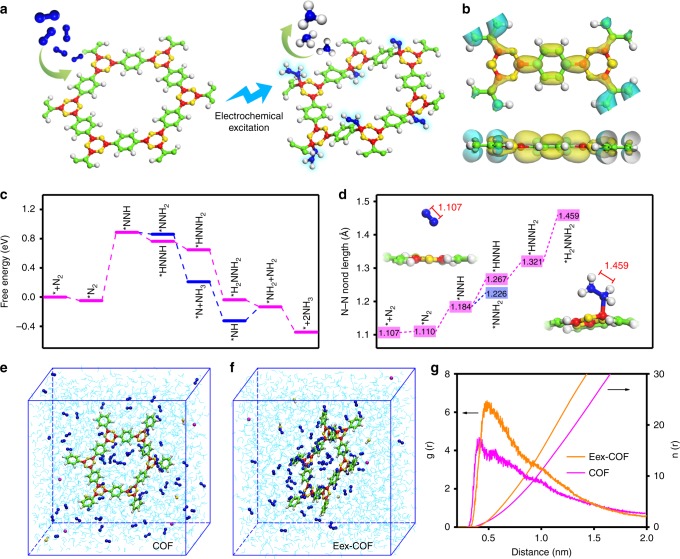
Computational studies. **a** Schematic illustration of the electrochemical excitation of COF. **b** The calculated charge distribution of COF. **c** Free energy diagrams and **d** changes of N–N bond length for ammonia synthesis. Molecular dynamics simulation snapshots of **e** COF and **f** Eex-COF. **g** Radial distribution function (RDF) and integrated RDF of nitrogen molecules around different models. The red, green, blue, yellow, and gray spheres represent B, C, N, O, and H atoms, respectively

In this article, we demonstrate that electrochemical excitation of boron-rich COFs could facilitate the catalyst accessibility to N_2_ and start up efficient NH_3_ synthesis under ambient conditions (Fig. 1a). Density functional theory (DFT) calculations suggest a superior electronic structure for N_2_ absorption and greatly reduced energy barrier of N_2_ dissociation over the boron atoms, and they are, therefore, easy to bond with nitrogenous species when suitable potential is applied. The formation of B–N bonds causes significant distortion of the COFs’ lattice planes, and the reconstruction greatly enhances the N_2_ adsorption toward the COFs networks, as illustrated by molecular dynamics (MD) simulations. The localized high N_2_ concentration would promote the collision probability of N_2_ molecules and the boron sites, so that to facilitate the whole reaction. Careful characterization of the proof-of-concept materials confirms that the COFs exhibit an evident transformation from crystalline to amorphous phase, as well as the appearance of B–N bond along with electrochemical excitation, which are also verified by in situ X-ray powder diffraction (XRD) and in situ Raman measurements. Since the linear sweep voltammetry (LSV) responses show facilitated NRR ability due to the above transformations, we ultimately conclude that the COFs undergo a virtuous cycle of more excitated sites and stronger N_2_ affinity until the whole system reaches the high-point ready for efficient NRR. By quantifying the NH_3_ produced, a high Faradaic efficiency of 45.43% is observed.

## Results

### Theoretical calculations of the boron-rich COFs

The COFs formed by self-condensation of 1,4-benzenediboronic acid (BDBA) as monomer were chosen as the study model, and named as COF for short in this paper. The catalytic activity was preliminary investigated by DFT computations. As shown in Fig. 1b, the lowest unoccupied molecular orbital (LUMO; yellow) and the highest occupied molecular orbital (HOMO; cyan) illustrate an obviously localized electron distribution, with the positive charge mainly locates around the boron atoms, indicating that the boron in B_3_O_3_ as Lewis acid is the potential active site for N_2_ (a weak Lewis basic gas) fixation. Since the electron deficiency structure of boron sites provides a unique environment for N_2_ adsorption, we further evaluated the subsequent NRR effect of different mechanisms from the thermodynamic perspective by means of first-principles calculations (Supplementary Fig. 1). The reference potential is set to be that of the normal hydrogen electrode (NHE), and the Gibbs free energy is given at U = 0 V versus (vs.) NHE. Upon energy minimizing, the NRR reaction on boron sites prefer to follow the associative alternating pathway with only one energy hill presents at the N_2_ activation step (Fig. 1c). This first N–N bond cleavage process usually requires a large energy input of 4.26 eV due to the inert nature of N_2_, and is thus widely accepted as the rate-determining step (RDS). While, in this model, the energy barrier of RDS is greatly reduced to 0.93 eV compared with the case without the catalyst. After N_2_ activation, the Gibbs free energy exhibits a step-by-step downhill trend accompanied with an obvious N–N bond elongation from 1.110 to 1.459 Å (Fig. 1d). In brief, the COF could serve as efficient catalyst for NRR from the thermodynamic view. Interestingly, an unusual phenomenon was noticed in the DFT models that the lattice planes of COF were obviously distorted when the active boron sites are bonded with nitrogenous species. Considering that such transformation could be excitated by electrochemical process when sufficient voltage was applied, more efforts should be made to give a deeper investigation into its actual influences.

Since the dynamic performance is another key point for heterogeneous catalysis system, we carried out the MD simulations to explore whether the aforementioned phenomenon favors the N_2_ diffusion in the catalyst. According to the DFT results, the adsorbed N_2_ (*N_2_) would be the majority among the bonded nitrogenous species as it exists before the only energy barrier. Thus, the electrochemically excitated COF (Eex-COF) model was set up by symmetrically placing *N_2_ at specific boron sites in every B_3_O_3_, and pristine COF was also calculated for comparison. 0.1 M KOH was chosen as the electrolyte to avoid the strong HER in acidic solution. In detail, N_2_-saturated alkaline solution served as the initial reaction system, and was set up by randomly placing N_2_, H_2_O, K^+^, and OH^−^ according to their molar concentration. The initial configuration was energy-minimized with the steepest descent algorithm, followed by short equilibration for 30 ps. Subsequently, 50 ns MD runs were performed under the NPT ensemble (298 K, 1 bar). As shown in Supplementary Fig. 2a, b, the Eex-COF exhibits an obvious distortion that in agreement with the DFT model. The snapshots (Fig. 1e, f), at the same time, illustrate a clear distinction of the two models that the N_2_ molecules tend to aggregate more compactly around the Eex-COF. To confirm this, radial distribution function (RDF) was calculated (Fig. 1g). Though the characterized peak of Eex-COF located at 0.48 nm from boron site is 0.06 nm farther than that in COF due to the block of *N_2_, the intensity is much higher, suggesting stronger N_2_ affinity to the structure. The integrated RDF also displays a much higher density of N_2_ molecules around the Eex-COF, which is in great accordance with the snapshots and RDF conclusion. The electrostatic and van der Waals (vdW) interactions of the two models and N_2_ molecules were further obtained by extracting the two energy groups from the MD trajectories (Supplementary Fig. 2c). Clearly, the vdW interaction is greatly enhanced after the excitation and plays a dominant part in the adsorption process. Considering the natures of N_2_ and the models, the facilitated N_2_ physisorption can be explained by the significantly increased London dispersion force (LDF)^29–31^. When significant distortion of COF happens, more atoms would interact with N_2_ from the same distance, resulting in much stronger LDF and thus greatly enhanced physisorption of N_2_^32,33^. A localized high N_2_ concentration would significantly benefit the gas diffusion and supply toward the active sites, so that is a prerequisite for efficient NRR. Thus, the electrochemically excitated structure would greatly benefit the whole NRR process.

### Synthesis and characterization of the Eex-COF/NC

**Fig. 2:**
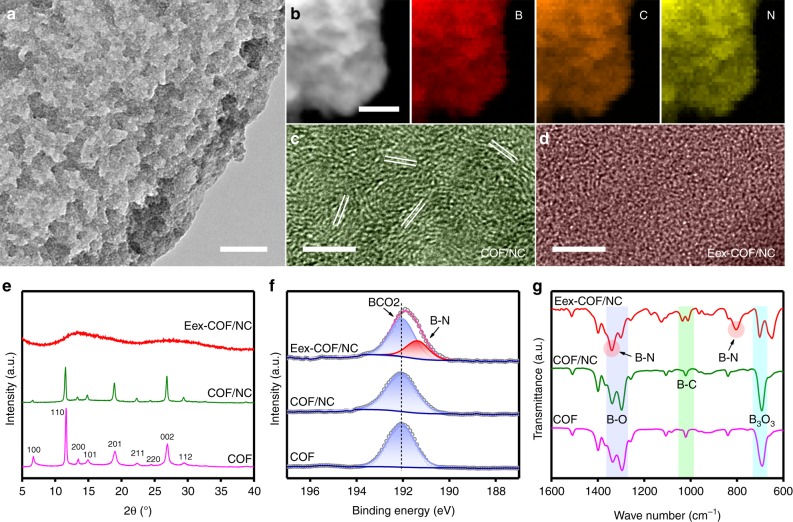
Physical characterization. **a** Transmission electron microscopy (TEM) image and **b** element mappings of Eex-COF/NC. High-resolution TEM images of **c** COF/NC and **d** Eex-COF/NC. Scale bars, **a** 200 nm; **b** 100 nm; **c** 5 nm and **d** 5 nm. **e** X-ray powder diffraction (XRD) patterns, **f** high-resolution B 1s spectra, and **g** Fourier-transform infrared (FTIR) spectra of different samples

To verify the theoretical conclusions, a series of proof-of-concept experiments were carried out. Considering the poor electrical conductivity of COF, conductive nitrogen-doped carbon nanosheet (NC, Supplementary Figs. 3 and 4) was fabricated as support to avoid such disadvantage. As demonstrated by X-ray photoelectron spectroscopy (XPS, Supplementary Fig. 5), graphitic-N and pyridinic-N are the main existence forms of nitrogen in NC. With the above two nitrogen species as study models, we carried out DFT calculations to explore the catalytic activity of the NC support (Supplementary Fig. 6). Clearly, the hydrogen evolution reaction (HER) activities of graphitic-N and pyridinic-N are rather weaker than COF, and the NRR activity is even worse with significantly higher energy barriers. Thus, its performance would not cause obvious influence on the subsequent analysis of COF. The NC was preoxidized and treated with BDBA in methanol solution to create nucleation sites for the growth of COF framework, and COF was then coated on it by molecular dehydration reaction of BDBA in mesitylene-dioxane solution via solvothermal reaction (COF/NC, Supplementary Fig. 7). The eletrochemically excitated COF/NC (Eex-COF/NC) was obtained by potentiostatic electrolyzing at −0.2 V vs. reversible hydrogen electrode (RHE) in N_2_-saturated 0.1 M KOH for 20 min. Pure COF was also synthesized for reference. It should be pointed out that the N_2_ was sufficiently purified before use to avoid the possible existence of NH_3_ or NO_x_ in all measurements in this work^13,34^ (Supplementary Figs. 8–10). The transmission electron microscopy (TEM) image of Eex-COF/NC (Fig. 2a) shows a homogeneous COF matrix over the substrate without any changes of surface morphology compared with COF/NC, with corresponding element mappings showing the uniform distribution of B, C, and N (Fig. 2b). Yet, the high-resolution TEM (HRTEM) images reveal an obvious transformation from crystalline (Fig. 2c) to amorphous phase (Fig. 2d). The XRD pattern of COF/NC shows all the characteristic peaks of COF, which is align with the previous report^35^, whereas the diffractions disappear after electrochemical excitation (Fig. 2e), which is consistent with the amorphous phase. This can be further confirmed by the cross-polarization magic angle spinning nuclear magnetic resonance (CP/MAS NMR) responses. The dramatical broaden and weaken of the ^13^C NMR spectra (Supplementary Fig. 11a) indicate a completely anisotropic chemical environment in Eex-COF/NC, and the shift of ^11^B NMR signal (Supplementary Fig. 11b) suggests that the bonding environment of boron might be changed. The surface chemistry of different samples was subsequently analyzed by XPS (Supplementary Fig. 12). As shown in Fig. 2f, a new peak representing B–N is captured in the high-resolution B 1s spectra of Eex-COF/NC. Simultaneously, same variation is also discovered in Fourier-transform infrared (FTIR) spectrum with the appearance of two new peaks located at 815 and 1370 cm^−1^ assigning to B–N^36^ (Fig. 2g). The strong peak appeared in the range of 620–680 cm^−1^ in Eex-COF/NC is ascribed to the out of plane phenyl ring deformation for benzene ring due to the significant lattice distortion, as well as C–H deformation resulting from the HER^35,37,38^. In the region highlighted in light green, the B–C stretching at 1023 cm^−1^ in COF/NC shifts to lower wavenumber in Eex-COF/NC due to the bonding between boron and nitrogen after electrochemical excitation^39^, and the new peak located at about 1040 cm^−1^ can be ascribed to benzyl ring deformations^40^ in COF. The results above are combined to elucidate that, the bonding of nitrogenous species on boron contributes to the distortion of the lattice, and provides direct evidence that the boron atoms serve as the NRR active sites.

### In situ characterizations and eletrochemical responses

**Fig. 3:**
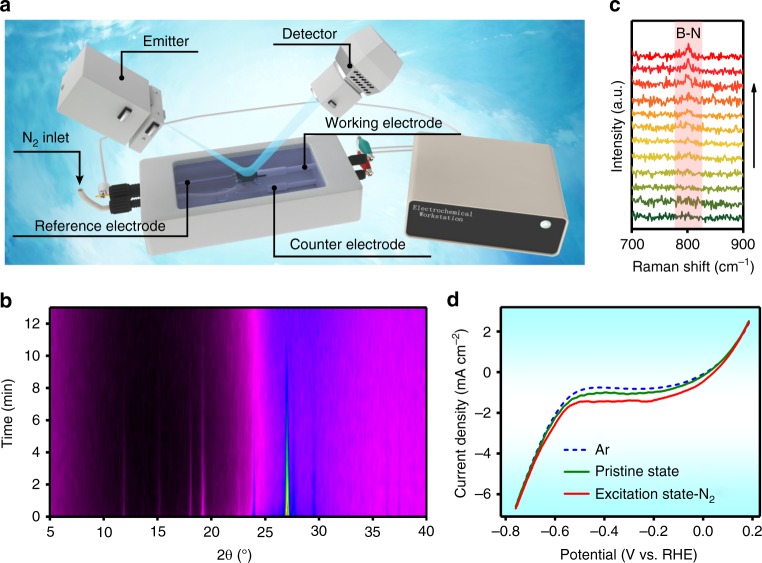
In situ characterizations and electrochemical responses. **a** Schematic illustration of the tailor-made electrolytic cell for in situ characterizations. **b** In situ XRD intensity map, and **c** in situ Raman spectra of the transformation from COF/NC to Eex-COF/NC. **d** Linear sweep voltammetry (LSV) curves of different states through excitation

To give a real-time observation of the unique conversion, in situ characterizations were employed in a tailor-made electrolytic cell (Fig. 3a). In situ XRD experiment was first implemented to examine the crystallinity change. As shown in Fig. 3b and Supplementary Fig. 13, legible characteristic peaks are detected in the pristine COF/NC electrode. While, all diffractions weakened gradually when a constant excitation voltage of –0.2 V vs. RHE was applied, and completely vanished at about 12 min. However, the characteristic peaks of COF remain almost unchanged in the same period of time when replacing N_2_ with Ar (Supplementary Figs. 14 and 15), suggesting that the transformation of COF from crystalline to amorphous phase is associated with the NRR process. Subsequently, we captured the in situ Raman spectra under the same operating conditions. When using N_2_ as gas supply (Fig. 3c), an obvious band at about 800 cm^−1^ attributed to B–N stretching vibration^41^ gradually appeared as the excitation time passed, whereas no such transformation was detected under Ar (Supplementary Fig. 16). These phenomena again imply that the lattice distortion of COF is due to the chemisorption of nitrogen or nitrogenous intermediates on the active boron sites. Now that such transformations above are proved to be beneficial for N_2_ aggregation around the COF by theoretical calculations, it is necessary to verify if they profit the actual NRR process. Thus, LSV curves were collected in both N_2_- and Ar-saturated 0.1 M KOH electrolyte (Fig. 3d and Supplementary Fig. 17). Expectedly, the LSVs exhibit a tendency that the current density gap between N_2_ and Ar environment increases from the pristine state to transition state, and then to the excitation state, illustrating the intensified reaction activity, accompanied by the positively shifted onset potential. That means, the COF/NC undergoes a warming-up procedure through electrochemical excitation and finally reaches an optimum status with dramatically high activity for efficient NRR. Combining the theoretical and experimental results, we can explain the mechanism and advantage for electrochemical excitation of COF as follows: the superior electronic structure and reduced energy barrier of RDS makes the boron sites in COF easy to bond with nitrogenous species under electrochemical condition; the resultant amorphous structure has stronger affinity toward the N_2_ molecules that could facilitate the molecular collision in the reaction to excite more boron atoms; the reconstruction induced by the enriched excitated boron sites further enhance the adsorption of N_2_ toward the COF networks. Such virtuous cycle continuously proceed until the whole system attains the high-point for NRR (Supplementary Fig. 18).

### Electrocatalytic nitrogen reduction performance of Eex-COF/NC

**Fig. 4:**
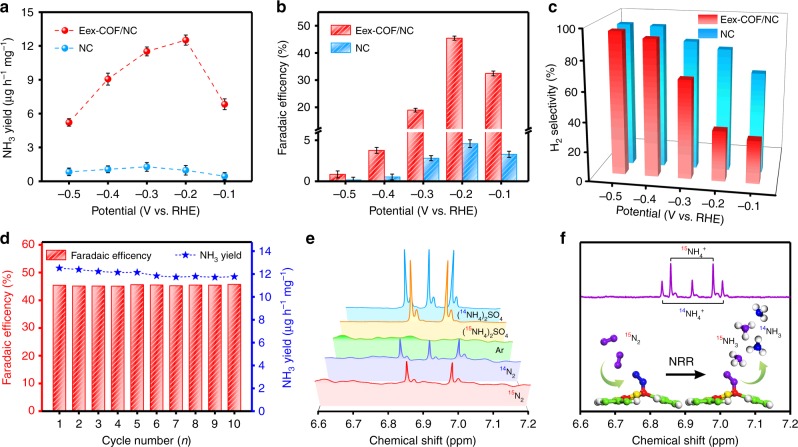
Electroreduction of N_2_ to NH_3_ at ambient conditions. **a** NH_3_ yield rates, **b** corresponding Faradaic efficiencies, and **c** H_2_ selectivity of Eex-COF/NC and NC. The error bars correspond to the standard deviations of measurements over three separately prepared samples under the same conditions. **d** The NH_3_ production performance in durability test of Eex-COF/NC. **e** ^1^H nuclear magnetic resonance (NMR) spectra of the NRR products using the same isotope feeding gas for excitation and NRR. **f** ^1^H NMR spectrum of the NRR products using ^14^N_2_ for excitation and ^15^N_2_ for NRR. The red, green, blue, purple, yellow, and gray spheres represent B, C, ^14^N, ^15^N, O, and H atoms, respectively

Quantified study of Eex-COF/NC of the NRR activity was then performed in 0.1 M KOH using a gas-tight two-compartment cell through chronoamperometry measurement (Supplementary Figs. 19 and 20). NH_3_ and N_2_H_4_ as possible products were both examined (Supplementary Figs. 8 and 21–23), whereas only NH_3_ was obtained in this work. Figure 4a, b shows the NH_3_ yield rate and corresponding Faradaic efficiency under different given potentials. Strikingly, the Faradaic efficiency and NH_3_ yield rate of Eex-COF/NC both reach the maximum value of 45.43% and 12.53 μg h^−1^ mg^−1^, respectively, at −0.2 V vs. RHE, which is a record-high activity among the metal-free catalysts to date (Supplementary Table 1). In contrast, the NC substrate exhibits an inferior NRR activity so that its contribution to the NH_3_ synthesized in Eex-COF/NC is almost neglectable. Compared with NC, in which the majority of electrons tends to offer up the HER, the HER is greatly reduced in the voltage range from −0.1 to −0.3 V vs. RHE after the electrochemical excitation of COF/NC (Fig. 4c), and more electrons are captured by NRR. On the other hand, the Eex-COF/NC can keep the superior performance almost unchanged under 10 successive cycles of NRR electrolysis (Fig. 4d), with the morphology and chemical construction remain constant (Supplementary Figs. 24–28), revealing the robust stability. Control experiments (Supplementary Fig. 29) exclude the probable existence of NH_3_ in the pristine feeding gas and the NRR ability of the carbon paper (CP), so that all the NH_3_ detected is confirmed to be produced by Eex-COF/NC. Furthermore, the ^15^N isotope labeling experiments (Supplementary Figs. 30–31) were also conducted to examine the N source of the obtained NH_3_. The ^15^N_2_ gas was also sufficiently purified before test (Supplementary Fig. 32). The produced ^15^NH_3_ is confirmed to be completely from the electrochemical NRR process (Supplementary Fig. 33), and the NH_3_ yield rate and corresponding Faradaic efficiency are in great accordance with the data obtained under ^14^N_2_ (Supplementary Fig. 34), thus making the experimental results reliable. As can be seen in Fig. 4e, when using ^15^N_2_ as feeding gas for both the electrochemical excitation and chronoamperometry measurement, only a doublet signal representing ^15^NH_4_^+^ is detected in the ^1^H NMR spectra, declaring the total N source from the feeding gas rather than any nitrogen species in NC. In addition, argon control experiments were also conducted at each given potential (Supplementary Fig. 35a). As demonstrated in ultraviolet-visible (UV-vis) spectra and ^1^H NMR analysis (Supplementary Figs. 35b and 36), no NH_3_ was detected when Ar was supplied, confirming that the nitrogen in NC substrate is not active enough to join the electrochemical conversion to NH_3_. Notably, we then used ^14^N_2_ for the excitation process and ^15^N_2_ for the subsequent NRR test, with both ^14^NH_4_^+^ and ^15^NH_4_^+^ observed in the evaluated products (Fig. 4f). Therefore, the pre-bonded nitrogen through electrochemical excitation are proved to be involved in the following conversion to NH_3_. Considering that there is no difference between the chemical constructions of Eex-COF/NC before and after the NRR test, the catalytic mechanism of Eex-COF/NC is inferred to be a continuous nitrogen substituting process, which is stable and maintains the catalyst with high nitrogen affinity that greatly favors the NRR.

## Discussion

In summary, we demonstrate a seminal discovery that electrochemical excitation of COF is able to promote the catalyst accessibility to N_2_ and launch highly efficient NRR under ambient conditions. DFT calculations show a beneficial charge distribution and reduced energy barrier of RDS over boron sites in the COF, so that they are facile to bond with nitrogenous species under electrochemical condition. The amorphous structure caused by the fixation of nitrogenous species is confirmed to have stronger affinity to the N_2_ molecules by MD simulations, which could enhance the collision frequency of the N_2_ molecules toward the boron sites in the NRR process and thus benefit the whole reaction system. In situ XRD, in situ Raman and careful experiments are combined to verify the theories, and we finally draw a conclusion that the COF undergoes a continuous warming-up procedure until the whole system attains an optimum status with strikingly high activity for efficient NRR. As a proof of concept, the target catalyst (Eex-COF/NC) tested in an actual NRR process achieves an outstanding Faradaic efficiency as high as 45.43%, outperforming all the metal-free catalyst to date to the best of our knowledge.

## Methods

### Computational method and model

The first-principles calculations were conducted using Cambridge Sequential Total Energy Package (known as CASTEP). The electron–electron interaction was described using the exchange-correlation function under the generalized gradient approximation (GGA) with norm-conserving pseudopotentials and Perdew-Burke-Ernzerhof functional. In total, 750 eV was used as the energy cutoff. Several parameters were considered, including energy tolerance of 5.0 × 10^−7^ eV per atom, a force tolerance of 0.01 eV Å^−1^ and maximum displacement of 5.0 × 10^−4^ Å. No constraints were set for any atom in the models, which is allowed to relax to the minimum in the enthalpy. The vacuum space along the *z* direction was set to be 15 Å.

Adsorption energy Δ*E* of A group on the surface of substrates was defined as:

1 $$\Delta E = E_{ \ast {\mathrm{A}}} - \left( {E_ \ast + E_{\mathrm{A}}} \right)$$

where *A and * denote the adsorption of A group on substrates and the bare substrates, *E*_A_ denotes the energy of A group.

Gibbs free energy change (Δ*G*) of each chemical reaction was calculated by:

2 $$\Delta G = \Delta E + \Delta ZPE - T\Delta S + \Delta G_{\mathrm{U}} + \Delta G_{{\mathrm{pH}}}$$

where *E*, *ZPE*, *T*, and *S* denote the calculated total energy, zero point energy, temperature, and entropy, respectively. Δ*G*_U_ = −e*U* (*U* is the potential measured against NHE) and Δ*G*_pH_ = −*kBT*ln(10) × pH. Here, *T* = 300 K and pH = 13 are considered.

MD simulations were carried out with Gromacs (v5.0.5). One COF molecule was immersed in KOH/N_2_ solution, which was composed of 60 N_2_ molecules, 4 K^+^, 4 OH^−^, and 2000 water molecules. The Eex-COF model was constructed by placing one *N_2_ at specific boron site in every B_3_O_3_ ring in COF.

The Universal force field (UFF)^42^ was adopted to the COF model. The topology file of COF was generated by the OBGMX program^43^, which includes the bonded (bond stretching, angle bending, and dihedral torsion) and nonbonded (interatomic van der Waals interactions represented by Lennard Jones model) parameters from UFF, while the partial charges were obtained from Zheng et al.^44^. Water, N_2_, and KOH were represented by the SPC/E model^45^, TraPPE model^46^ and OPLS-AA^47^ force field, respectively. Cross interactions between unlike atom pairs were calculated with the standard Lorentz-Berthelot combination rules. Short-range interactions were truncated at 1.2 nm, while the long-range coulombic interactions were calculated with the particle mesh Ewald algorithm with a Fourier spacing of 0.12 nm. Leapfrog algorithm was used to integrate the equations of motion with a time step of 1 fs.

Energy-minimization was first conducted with the steepest descent algorithm, followed by isothermal-isobaric equilibration at 298 K and 1 bar for 50 ps. Then, 50 ns MD runs were carried out under the *NPT* ensemble at 298 K and 1 bar, with Nose-Hoover thermostat and Parrinello-Rahman barostat controlling the system temperature and pressure, respectively. 3-D periodic boundary conditions were applied throughout simulations.

### Catalyst synthesis

NC was first prepared. Pyrrole (1 mL) was added to deionized water and treated by ultrasonic vibration to obtain a uniform suspension. Sodium chloride and zinc chloride with a molar ratio of 4:1, and 1 M HCl (1 mL) were successively dissolved into the above solution. The solution was cooled down to 0 °C in a low-temperature cooling system, followed by dropwise adding of (NH_4_)_2_S_2_O_8_ solution to trigger the pyrrole polymerization. The reaction was kept stirring for 3 h, and the obtained solution was freeze-dried for 24 h. The obtained solid was annealed for 2 h at 700 °C with argon protection. The salt template and impurities were washed away using hydrochloric acid, water, and ethanol successively. Then, the obtained products were dried under vacuum at 60 °C for 12 h. To prepare COF/NC, the NC was oxidized with piranha solution (14 mL of 98% H_2_SO_4_ and 6 mL of 30% H_2_O_2_) under vigorous stirring for 24 h, and thoroughly washed with deionized water and dried. Then 10 mg oxidized NC was sonicated in 2 mL methanol in a pyrex tube, followed by adding 30 mg BDBA. The tube was sealed under nitrogen and annealed at 90 °C for 24 h. The obtained materials were thoroughly washed with methanol, and then with mesitylene/dioxane solution (1:1 in volume). The products and 50 mg BDBA were further dispersed in 2 mL mesitylene/dioxane solution without drying. The tube was flash frozen with liquid N_2_, evacuated and flame sealed. The reaction was kept at 120 °C for 72 h, and the product was washed with acetone and stabilized under vacuum at 100 °C overnight to obtain COF/NC. The Eex-COF/NC was prepared by potentiostatic electrolyze the COF/NC at –0.2 V vs. RHE in N_2_-saturated 0.1 M KOH for 20 min. Pristine COF was synthesized using the same method as COF/NC without using the NC as substrate.

### Physical characterization

The morphology was studied via field emission transmission electron microscope (FETEM, FEI Tecnai G2 F20 S-TWIN TMP) operating at 200 kV. Surface elemental analysis was performed on X-ray photoelectron spectroscopy (XPS, Kratos Axis Ultra Dld). The structure of the catalysts was characterized by X-ray powder diffraction (XRD, D8 Advance, Bruker), nuclear magnetic resonance measurements (NMR, Agilent 600 MHz) and Fourier-transform infrared spectra (FTIR, Tensor 27, Bruker).

### Cathode preparation

One microgram of catalyst was dispersed in 100 μL solution consisting of 90 μL ethanol and 10 μL Nafion solution (5 wt%), and sonicated for at least 1 h to obtain a homogeneous ink. The catalyst ink was loaded on carbon paper with area of 1 × 1 cm^2^ to obtain a mass loading of 1 mg cm^−2^, and dried in N_2_ atmosphere at 80 °C for 1 h.

### Nitrogen purification

High purity ^14^N_2_ (Messer Gas, Germany) and ^15^N_2_ (99 atom% ^15^N, Newradar Special Gas Co. Ltd., Wuhan, China) was purchased commercially. The nitrogen gas was flowed through acid and alkaline trap successively to remove the possible NH_3_ and NO_x_, and passed a drying tube to block the vapor before supplied into the electrochemical cell. The purified gas was continuously passed through 0.1 M KOH solution for a period of time, and the obtained solution was analyzed using spectrophotometric methods to ensure that no NH_3_ or NO_x_ resides.

### Detection of ammonia

NH_3_ was determined by the indophenol blue method^48^ with some modification. In detail, 2 mL of the treated electrolyte was mixed with 2 mL of 1 M NaOH solution containing sodium citrate and salicylic acid. Then, 0.2 mL of 1 wt% sodium nitroferricyanide and 1 mL of 0.05 M sodium hypochlorite solution were added into the solution. After standing in darkness for 3 h, the UV-Vis absorption spectrum was measured. The concentration of indophenol blue was determined using the absorbance at a wavelength of 655 nm. The concentration–absorbance curves were calibrated using standard ammonia chloride solution with a series of concentrations in 0.1 M KOH.

### Detection of NO_x_

NO_x_ was determined using N-(-1-naphthyl)-ethylenediamine dihydrochloride spectrophotometric method^49^ with some modification. Detailedly, 0.5 g sulfanilic acid was dissolved in 90 mL H_2_O and 5 mL acetic acid. Then, 5 mg n-(1-naphthyl)-ethylenediamine dihydrochloride was added and the solution was filled to 100 mL to obtain chromogenic agent. One milliliter of the treated electrolyte was mixed with 4 mL chromogenic agent and left in darkness for 15 min. The UV-Vis absorption spectrum was then measured at 540 nm. The concentration–absorbance curves were calibrated using standard sodium nitrite solution with a series of concentrations in 0.1 M KOH.

### In situ characterization

A tailor-made cell was customized for electrochemical measurements. The Pt wire and Ag/AgCl (4 M KCl) were used as the counter and reference electrode, respectively. N_2_- or Ar-saturated 0.1 M KOH was used as the electrolyte. The catalyst ink was prepared by dispersing 10 mg COF/NC in 9 mL ethanol and 1 mL Nafion solution (5 wt%) in an ultrasonic bath for at least 1 h. The ink was coated on carbon paper to be used as the working electrode. The in situ XRD measurements were recorded at the X-ray powder diffractometer (D8 Advance, Bruker). The in situ Raman measurements were conducted at the Raman spectroscopy (HR evolution, Horiba Jobin Yvon, France). The chronoamperometric method was conducted at −0.2 V vs. RHE by CHI660E electrochemical workstation (Shanghai Chenhua Instrument Co., Ltd). The working electrode was continuously bubbled by N_2_ through the in situ characterization.

### Electrochemical NRR measurements

The electrochemical measurements were conducted in a Nafion 211 membrane-separated two-compartment cell at room temperature. Both cathode chamber and anode chamber contained 30 mL of 0.1 M KOH electrolyte. Before measurements, the membrane was treated by hydrogen peroxide solution (5%) at 80 °C for 1 h and then deionized water at 80 °C for 1 h. Ag/AgCl (4 M KCl) was used as the reference electrode, and Pt foil was used as the counter electrode. For electrochemical NRR, potentiostatic tests were conducted in N_2_-saturated 0.1 M KOH, which was purged with purified N_2_ for 30 min before the measurement.

The potentiostatic tests were tested in 0.1 M KOH aqueous solution at different potentials including −0.1, −0.2, −0.3, −0.4, and −0.5 V vs. RHE. In order to avoid the volatilization of produced ammonia, another cell filled with 30 mL of 0.001 M H_2_SO_4_ as the gas absorption liquid was set at the end of the cathode cell. After NRR, the cathodic electrolyte and the absorption liquid were both collected and analyzed by chromogenic reactions for quantitative measurements. The total ammonia yield was the summation of that in 0.1 M KOH and 0.001 M H_2_SO_4_. Argon control electrochemical measurements were conducted using the same method with high purity argon as the gas supply.

### Detection of ammonia in gas absorption cell

Concentration of the absorbed ammonia in 0.001 M H_2_SO_4_ was determined using the same method as that in 0.1 M KOH.

### Detection of hydrazine

The hydrazine presented in 0.1 M KOH was estimated by the method of Watt and Chrisp^50^. A mixture of para-(dimethylamino) benzaldehyde (5.99 g), HCl (concentrated, 30 mL) and ethanol (300 mL) was used as a color reagent. Five microliters of the residual electrolyte after NRR potentiostatic test was removed from the electrochemical reaction vessel. Then, 5 mL of above prepared color reagent was added to the solution and stirred for 10 min at room temperature. The absorbance of the resulting solution was measured at a wavelength of 455 nm. The concentration–absorbance curves were calibrated using standard hydrazine monohydrate 0.1 M KOH solution with a series of concentrations. Concentration of produced hydrazine in 0.001 M H_2_SO_4_ was determined using the same method.

### Detection of hydrogen

The hydrogen evolution analysis was performed by a gas chromatography (Agilent 7890B, USA).

### Thermodynamic equilibrium potential

The standard potential of NRR at alkaline medium was calculated as below:

3 $${\mathrm{N}}_2({\mathrm{g}}) + 8{\mathrm{H}}_2{\mathrm{O}} + 6{\mathrm{e}}^ - \to 2{\mathrm{NH}}_4{\mathrm{OH}}({\mathrm{aq}}) + 6{\mathrm{OH}}^ - \quad \Delta G^{\mathrm{o}} = 426.38\,{\mathrm{kj}}\,{\mathrm{mol}}^{ - 1}$$

4 $$E^\circ = - \Delta G^o/nF = - 0.737\,{\mathrm{V}}\,{\mathrm{versus}}\,{\mathrm{Standard}}\,{\mathrm{Hydrogen}}\,{\mathrm{Electrode}}({\mathrm{vs}}.\,{\mathrm{SHE}})$$

where *F* is the Faraday constant (96,485 C mol^−1^) and *n* is the number of transferred electrons (6).

As for reaction conditions, the thermodynamic equilibrium potential was determined according to the Nernst equation, assuming 1 atm of N_2_ in the electrolyte.

5 $$E = E^\circ - RT/6F \times \ln [c^2({\mathrm{NH}}_4{\mathrm{OH}}) \times c^6({\mathrm{OH}}^ - )] + 0.059 \times {\mathrm{pH}}({\mathrm{vs}}.\,{\mathrm{RHE}})$$

where *F* is the Faraday constant (96,485 C mol^−1^), *T* is the temperature in Kelvin (298.15 K), *c*(OH^−^) is the hydroxide concentration (0.1 M), *R* is the gas constant (8.314 J mol^−1^ K^−1^), and pH is 13. Assuming a NH_4_OH concentration of 10^−6^ M in the electrolyte, the corresponding thermodynamic equilibrium potential is calculated to be 0.207 V vs. RHE.

### Ammonia yield rate and Faradaic efficiency

The yield rate and Faradaic efficiency of NH_3_ were calculated as below:

6 $${\mathrm{Faradaic}}\,{\mathrm{efficiency}}({\mathrm{NH}}_3) = [3F \times c({\mathrm{NH}}_3) \times V]/Q$$

7 $${\mathrm{Yield}}\,{\mathrm{rate}}({\mathrm{NH}}_3) = [17c({\mathrm{NH}}_3) \times V]/(t \times m)$$

where *m* is the loading mass of the catalysts, *t* is the electrolysis time (1 h), *V* is the volume of the electrolyte, *Q* is the total charge passed through the electrode, *F* is the Faraday constant (96,485 C mol^−1^), and *c*(NH_3_) is the measured ammonia concentration.

The Faradaic efficiency of hydrogen evolution was calculated as below:

8 $${\mathrm{Faradaic}}\,{\mathrm{efficiency}}\left( {{\mathrm{H}}_2} \right) = 2Fv\left( {{\mathrm{H}}_2} \right)Gp_0/RT_0i_{{\mathrm{total}}}$$

where *i*_total_ is the steady-state cell current, *G* is the gas flow rate (mL/min at room temperature and ambient pressure), *v*(H_2_) is the volume concentration of hydrogen in the exhaust gas from the electrochemical cell, *p*_0_ = 1.01 × 10^5^ Pa, *R* = 8.314 J mol^−1^ K^−1^.

### ^15^N isotopic labeling experiment

In the labeling experiment, ^15^N_2_ (99 atom% ^15^N, Newradar Special Gas Co. Ltd., Wuhan, China) was applied as the feeding gas. The electrolyte was taken out after electrolysis at −0.2 V vs. RHE for several hours. By adding 0.5 M sulfuric acid, the pH of the electrolyte was adjusted to 3. After concentration to 1 mL, 0.9 mL solution was taken out and 0.1 mL D_2_O was added in as an internal standard. (^15^NH_4_)_2_SO_4_ and (^14^NH_4_)_2_SO_4_ was used as the benchmark. The quantification of the produced NH_3_ was performed by ^1^H nuclear magnetic resonance measurement (Agilent 600 MHz, USA).

## Supplementary information

Supplementary Information

## Data Availability

The data that support the findings of this study are available from the corresponding author upon reasonable request.
