# Supplementary material for: Facilitating nitrogen accessibility to boron-rich covalent organic frameworks via electrochemical excitation for efficient nitrogen fixation
Source: Nat Commun. 2019 Aug 29;10:3898. doi: 10.1038/s41467-019-11846-x (PMC6715660; doi:10.1038/s41467-019-11846-x)
Supplement: Supplementary file 1 — Supplementary Information [file 41467_2019_11846_MOESM1_ESM.pdf]

# Supplementary Information

# Facilitating $N_2$ accessibility to boron-rich covalent organic frameworks via electrochemical excitation for efficient nitrogen fixation

**Liu et al.**

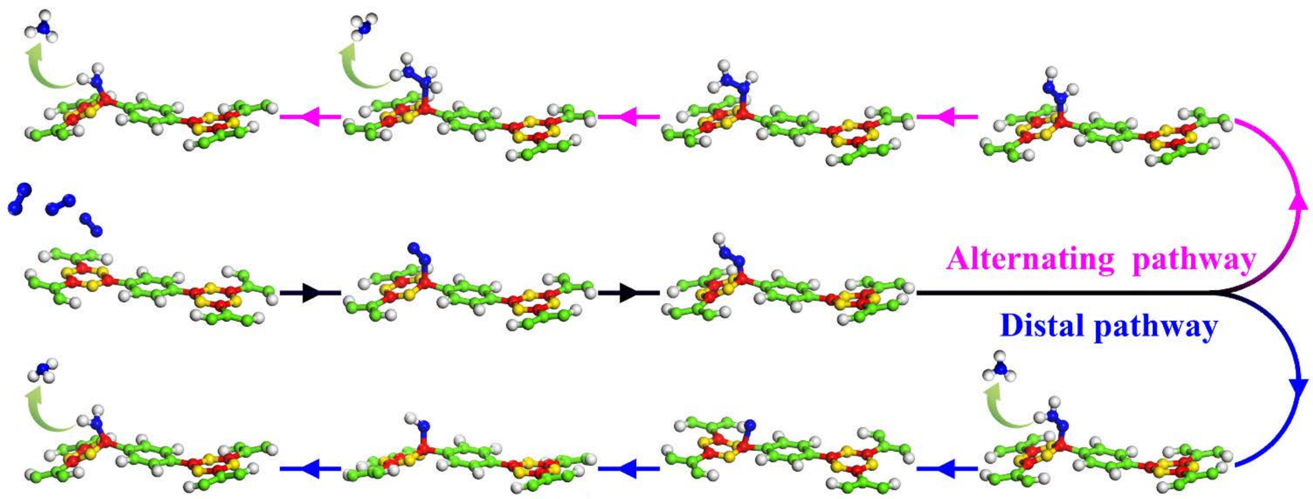


**Supplementary Figure 1.** The reaction pathways of COF for NRR. The red, green, blue, yellow and gray spheres represent B, C, N, O and H atoms, respectively.

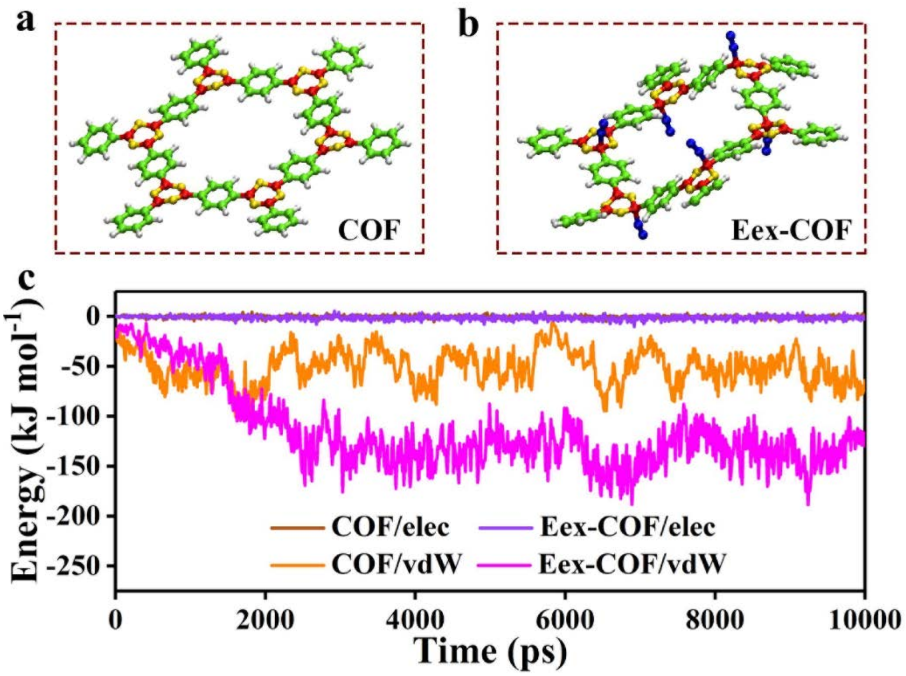


**Supplementary Figure 2.** The models of **a** COF and **b** Eex-COF in MD simulations. The red, green, blue, yellow and gray spheres represent B, C, N, O and H atoms, respectively. **c** Electrostatic and vdW interactions of COF and Eex-COF with the $N_2$ molecules.

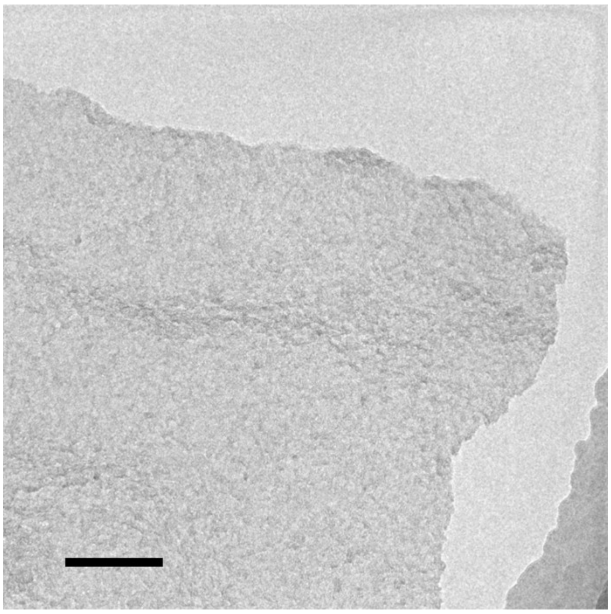

**Supplementary Figure 3.** TEM image of NC. Scale bar, 200 nm.

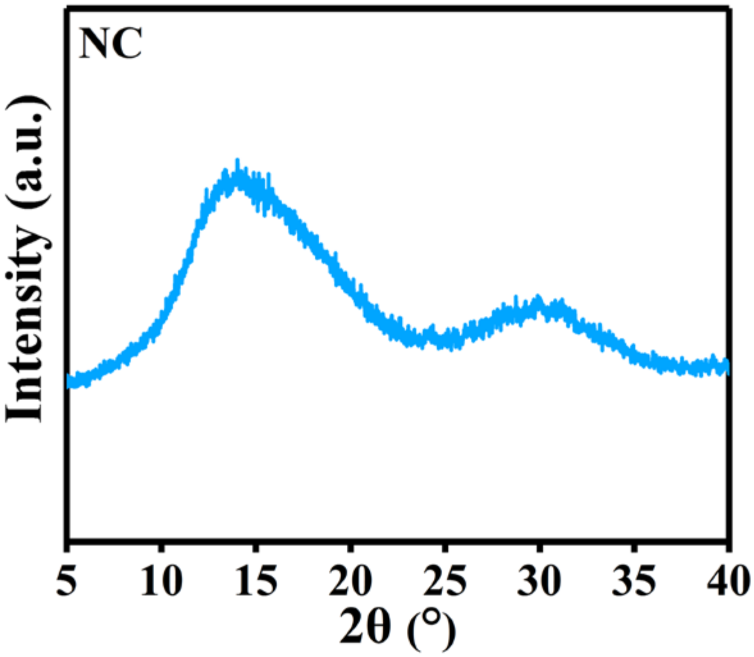

**Supplementary Figure 4.** XRD pattern of NC.

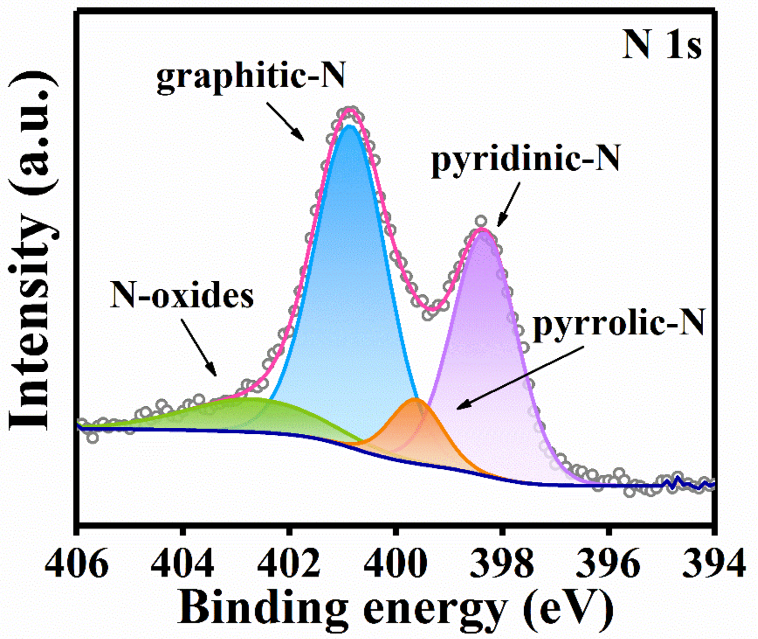


**Supplementary Figure 5.** High resolution N 1s spectrum of NC.

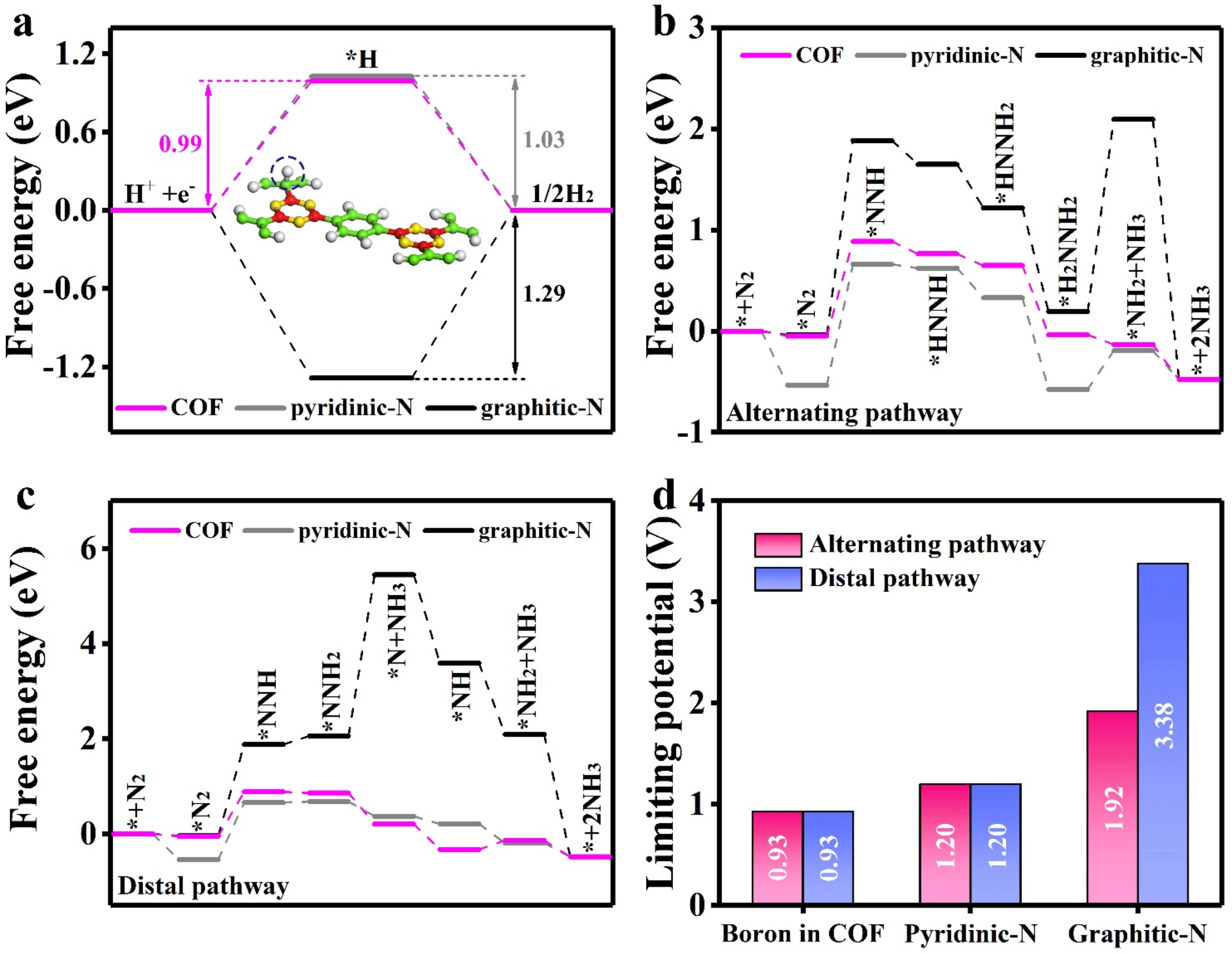


**Supplementary Figure 6.** Free energy diagrams of **a** HER, **b** NRR through the associative alternating pathway and **c** NRR through the associative distal pathway for different models. **d** Comparision of the NRR limiting potentials for different models. The red, green, yellow and gray spheres represent B, C, O and H atoms, respectively.

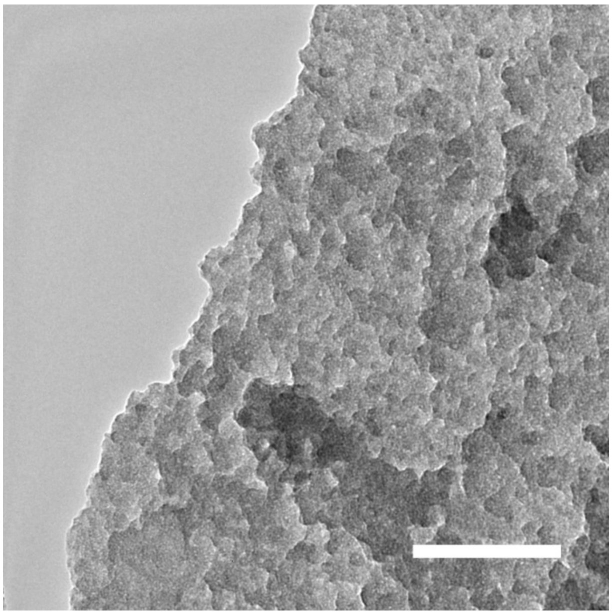

**Supplementary Figure 7.** TEM image of COF/NC. Scale bar, 200 nm.

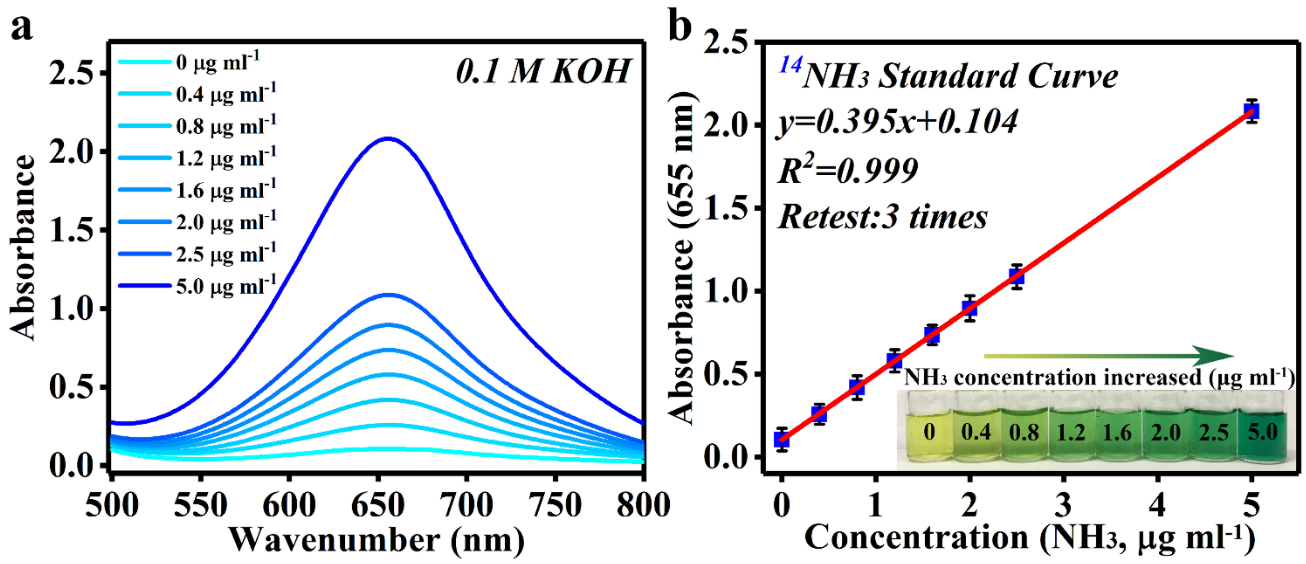


**Supplementary Figure 8. a** The UV-Vis absorption spectra and **b** corresponding calibration curves for the colorimetric $^{14}NH_3$ assay using the indophenol blue method in 0.1 M KOH. The error bars correspond to the standard deviations of measurements over three separately prepared samples under the same conditions.

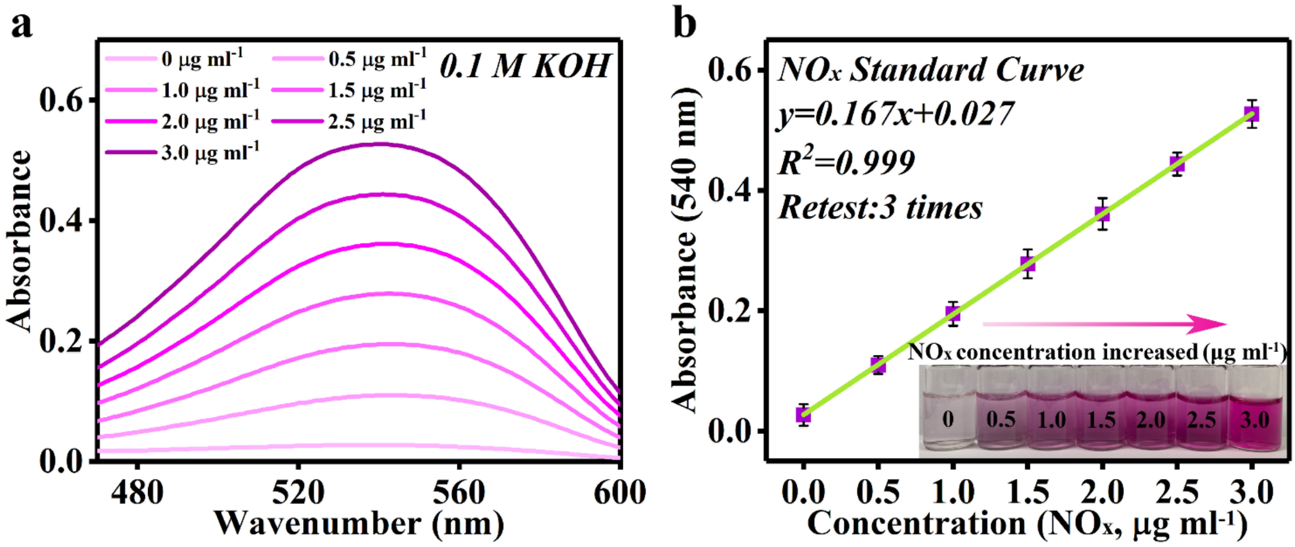


**Supplementary Figure 9. a** The UV-Vis absorption spectra and **b** corresponding calibration curves for the colorimetric $NO_x$ assay using the N-(-1-naphthyl)-ethylenediamine dihydrochloride spectrophotometric method in 0.1 M KOH. The error bars correspond to the standard deviations of measurements over three separately prepared samples under the same conditions.

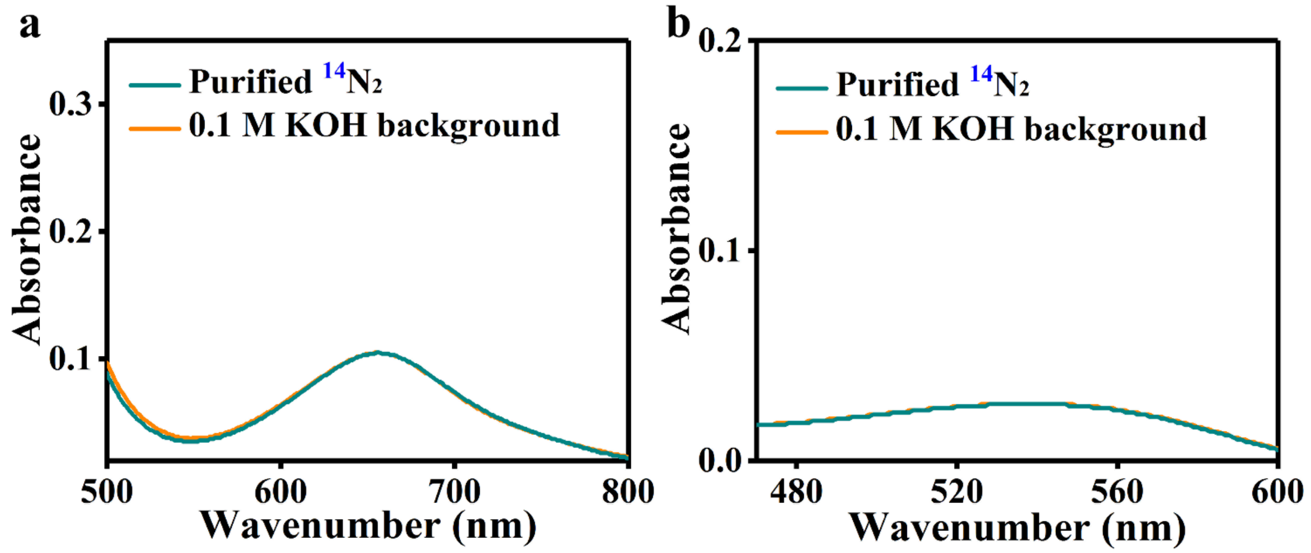


**Supplementary Figure 10.** The UV-Vis absorption spectra of the 0.1 M KOH background and the purified $^{14}N_2$ treated 0.1 M KOH solution using **a** indophenol blue method and **b** N-(-1-naphthyl)-ethylenediamine dihydrochloride spectrophotometric method. The results show that no $NH_3$ or $NO_x$ exists in the purified gas.

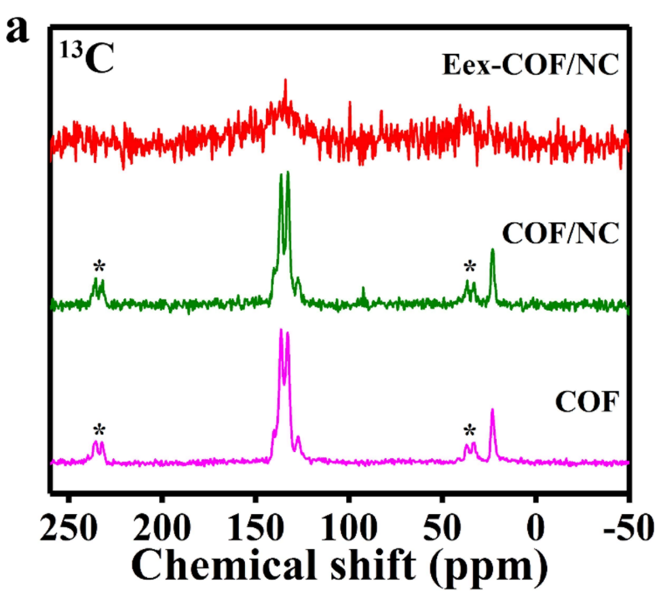


**Supplementary Figure 11. a** $^{13}$C and **b** $^{11}$B CP/MAS NMR spectra of different samples.

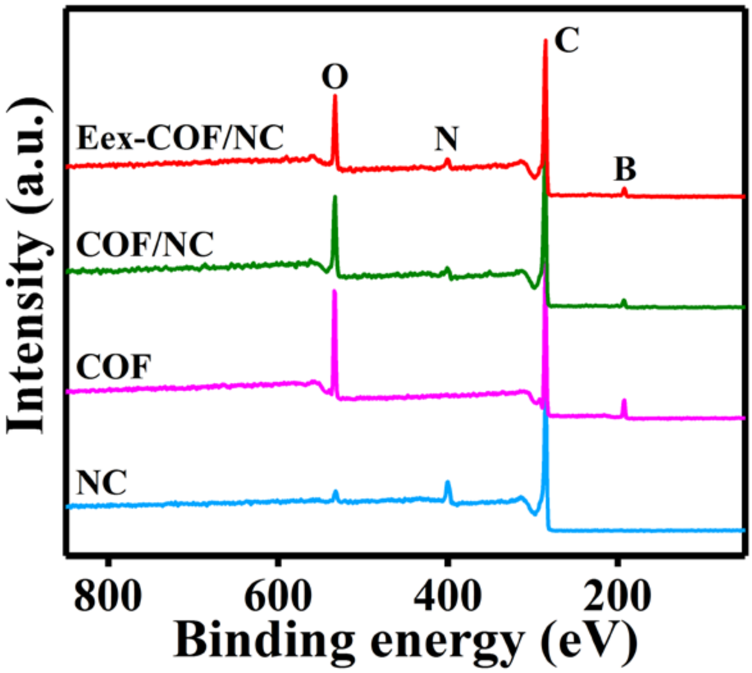


**Supplementary Figure 12.** The survey XPS spectra of different samples.

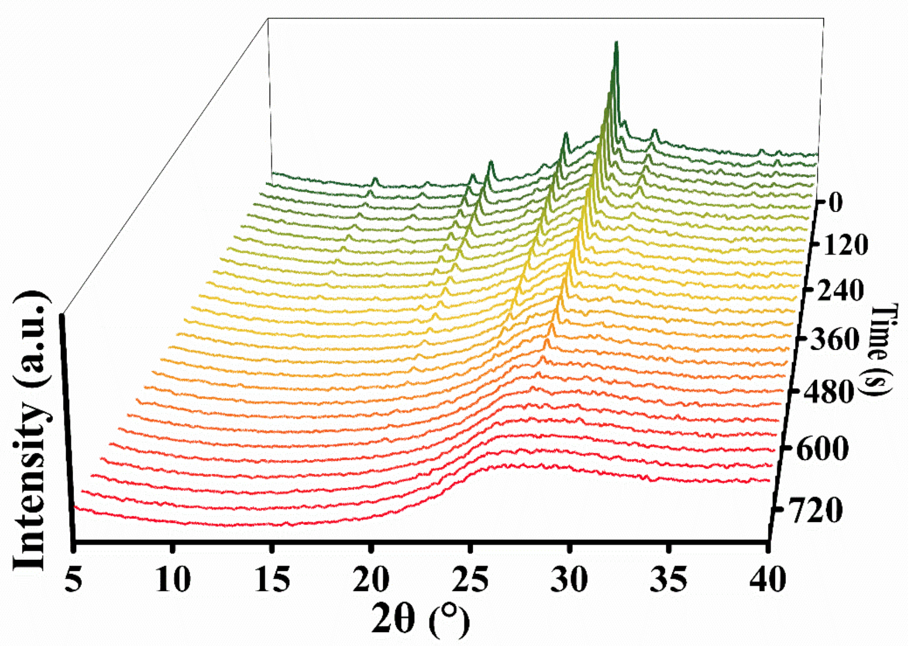


**Supplementary Figure 13.** In situ XRD patterns of the transformation from COF/NC to Eex-COF/NC during chronoamperometric operation at –0.2 V vs. RHE under $N_2$.

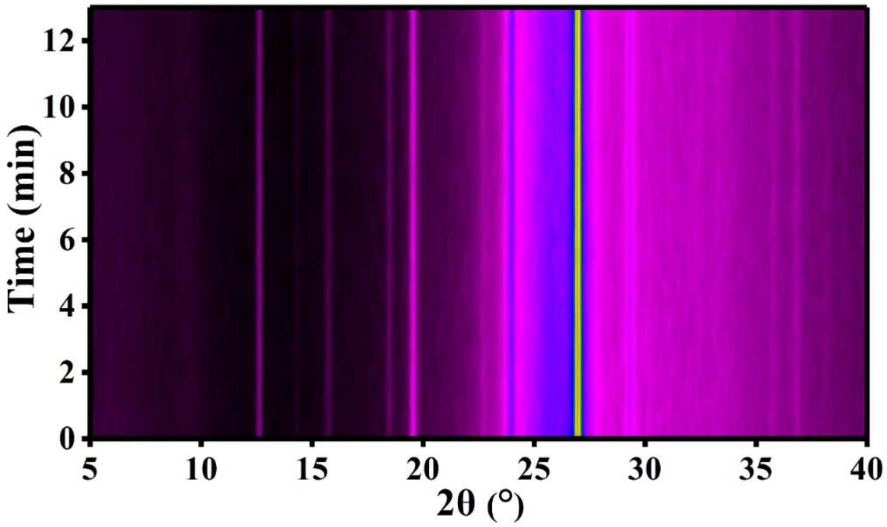


**Supplementary Figure 14.** In situ XRD intensity map of COF/NC during chronoamperometric operation at –0.2 V vs. RHE under Ar.

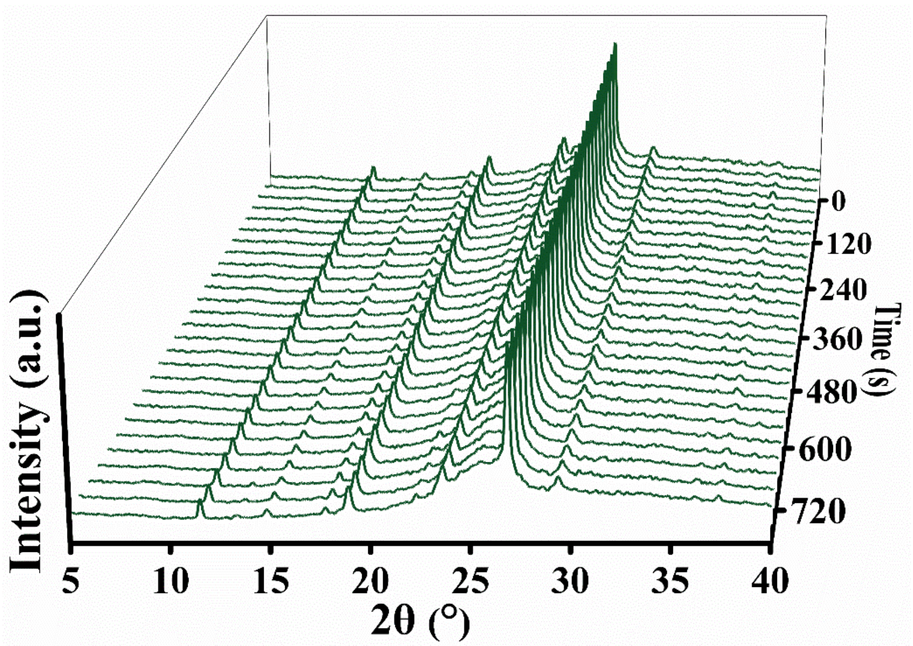


**Supplementary Figure 15.** In situ XRD patterns of COF/NC during chronoamperometric operation at –0.2 V vs. RHE under Ar.

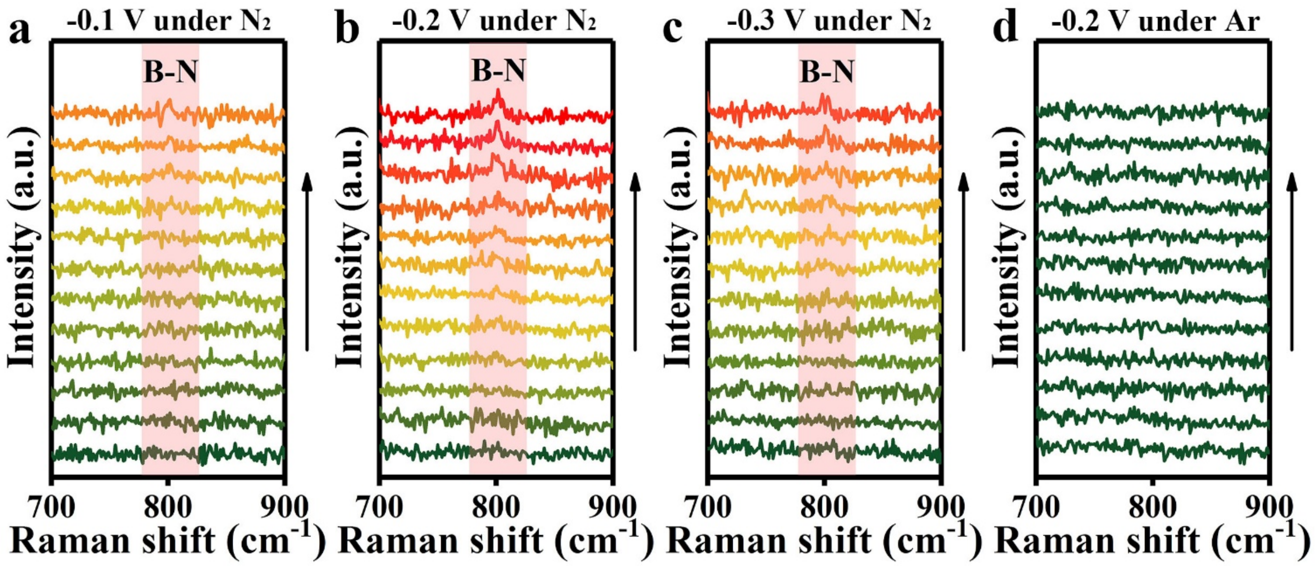


**Supplementary Figure 16.** In situ Raman spectra of COF/NC tested at **a** –0.1 V, **b** –0.2 V and **c** –0.3 V vs. RHE under $N_2$, and **d** –0.2 V vs. RHE under Ar. The results show the formation of B-N bonds under both –0.1 V and –0.3 V vs. RHE, indicating the occurrence of excitation. However, the transformations are slower and weaker than that measured at –0.2 V vs. RHE in the same testing period, and the extent increases in the order of –0.1 V < –0.3 V < –0.2 V vs. RHE. As a result, –0.2 V vs. RHE is supposed to be the optimal potential for the electrochemical excitation process.

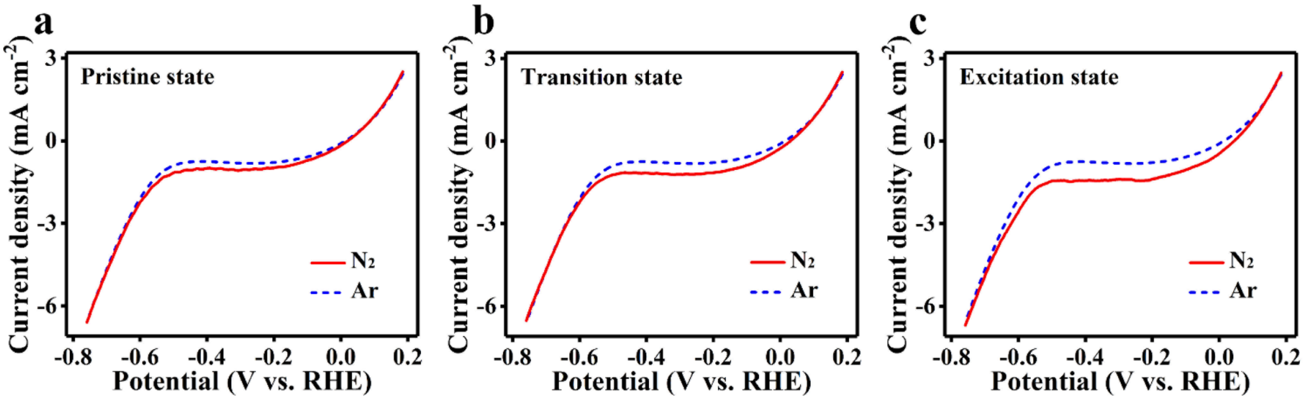


**Supplementary Figure 17.** LSV curves of **a** pristine state, **b** transition state and **c** excitation state.

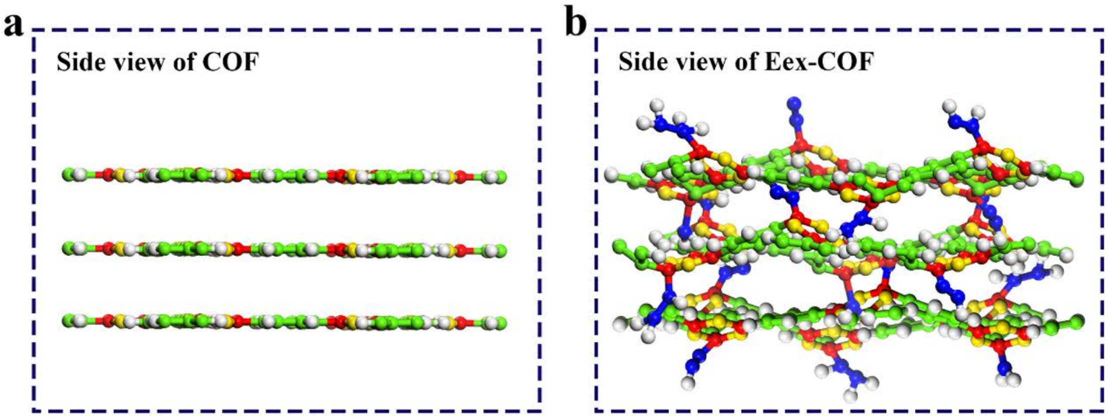


**Supplementary Figure 18.** Side views of **a** COF and **b** Eex-COF. The red, green, blue, yellow and gray spheres represent B, C, N, O and H atoms, respectively.

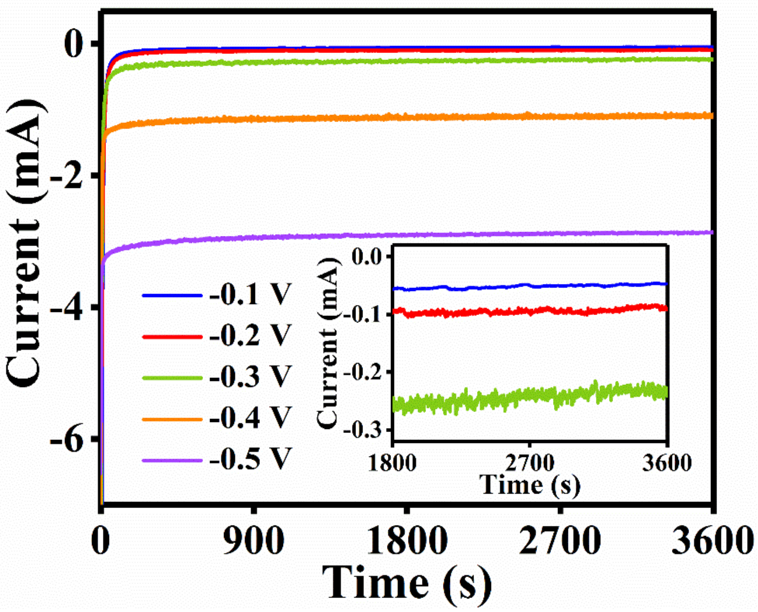


**Supplementary Figure 19.** Chronoamperometry results of Eex-COF/NC at the corresponding potentials, inset: enlarged view of the curves from –0.3 to –0.1 V vs. RHE.

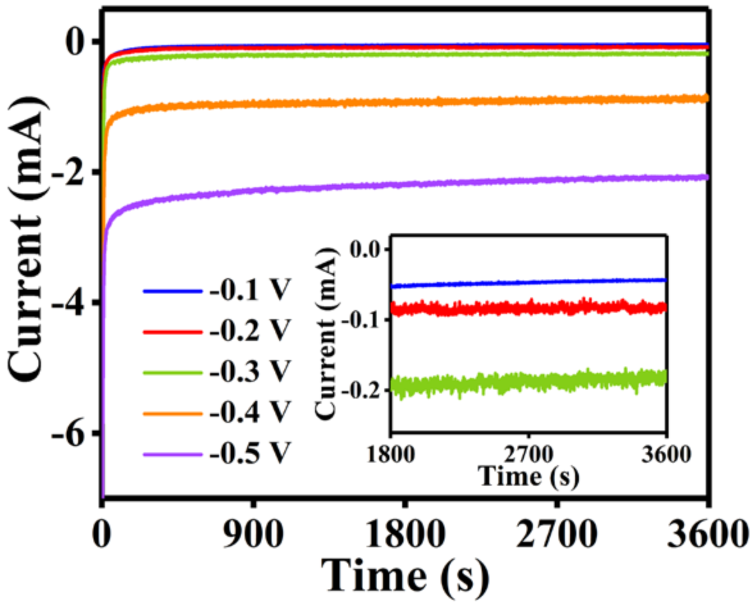


**Supplementary Figure 20.** Chronoamperometry results of NC at the corresponding potentials, inset: enlarged view of the curves from –0.3 to –0.1 V vs. RHE.

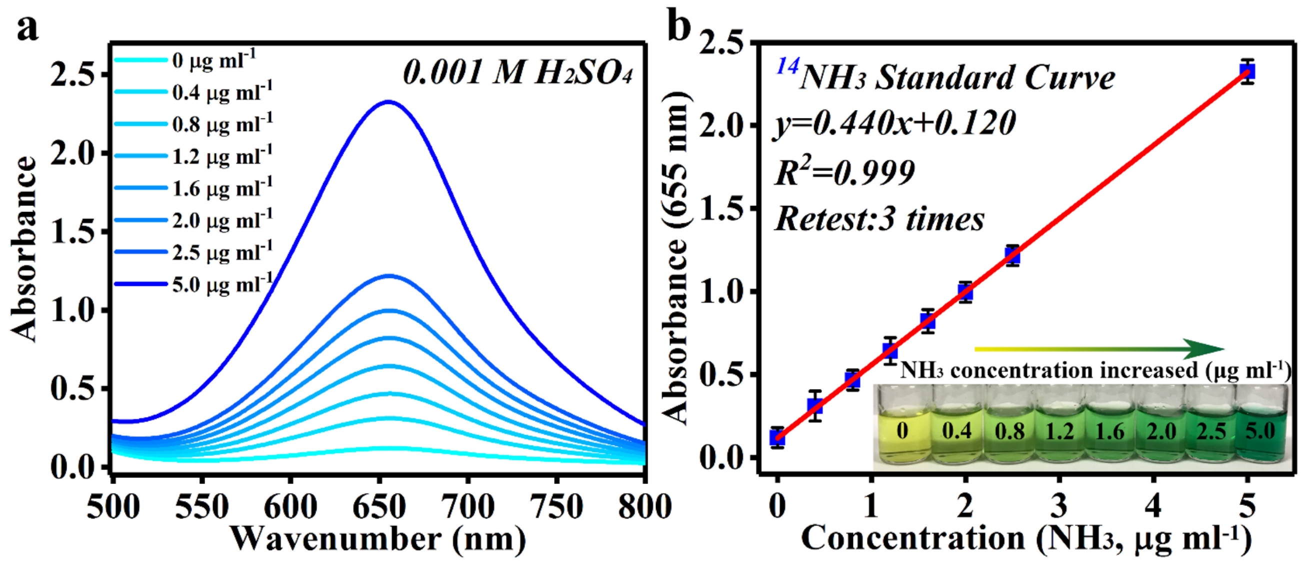


**Supplementary Figure 21. a** The UV-Vis absorption spectra and **b** corresponding calibration curves for the colorimetric $^{14}NH_3$ assay using the indophenol blue method in 0.001 M $H_2SO_4$. The error bars correspond to the standard deviations of measurements over three separately prepared samples under the same conditions.

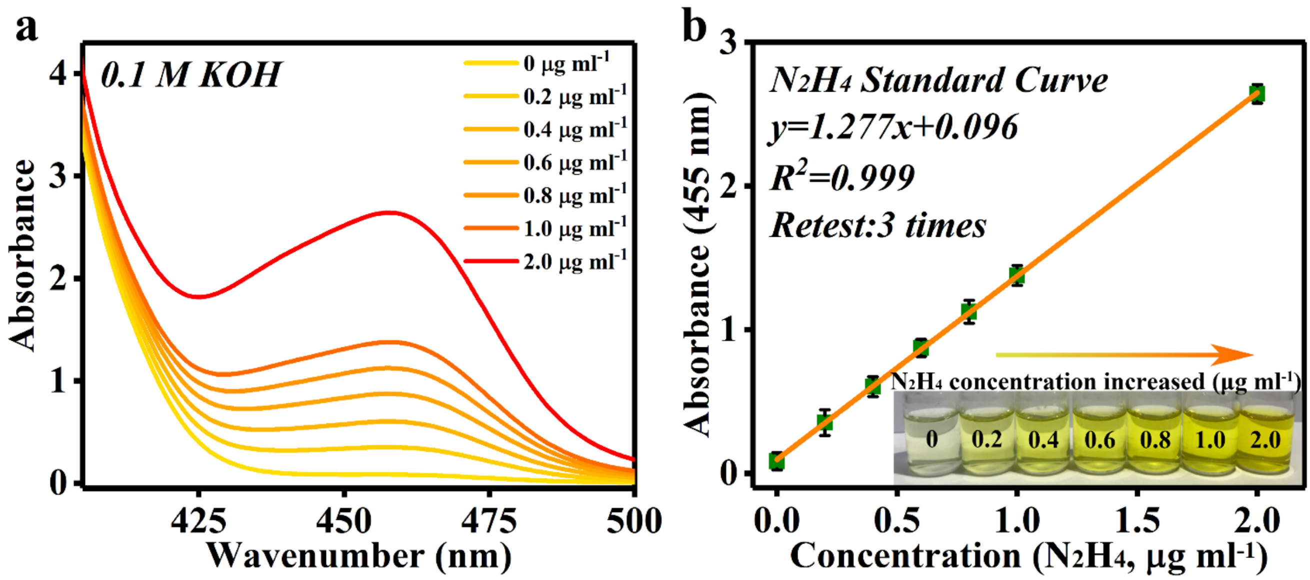


**Supplementary Figure 22.** **a** The UV-Vis absorption spectra and **b** corresponding calibration curves for the colorimetric $N_2H_4$ assay using the Watt and Chrisp method in 0.1 M KOH. The error bars correspond to the standard deviations of measurements over three separately prepared samples under the same conditions.

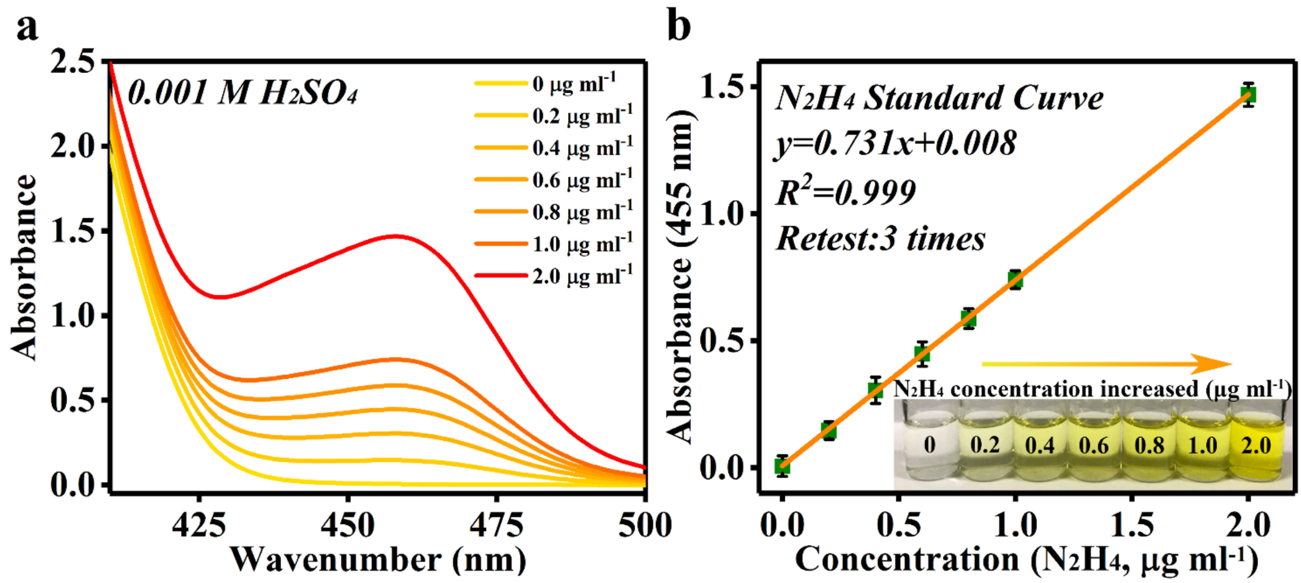


**Supplementary Figure 23. a** The UV-Vis absorption spectra and **b** corresponding calibration curves for the colorimetric $N_2H_4$ assay using the Watt and Chrisp method in 0.001 M $H_2SO_4$. The error bars correspond to the standard deviations of measurements over three separately prepared samples under the same conditions.

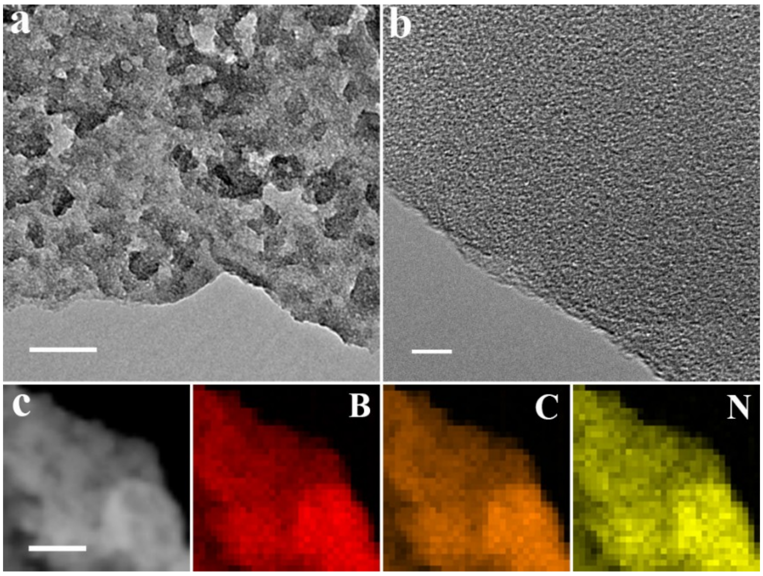

**Supplementary Figure 24.** **a** TEM image, **b** HRTEM image and **c** corresponding element mappings of Eex-COF/NC after NRR electrolysis. Scale bars, **a** 100 nm; **b** 5 nm and **c** 100 nm.

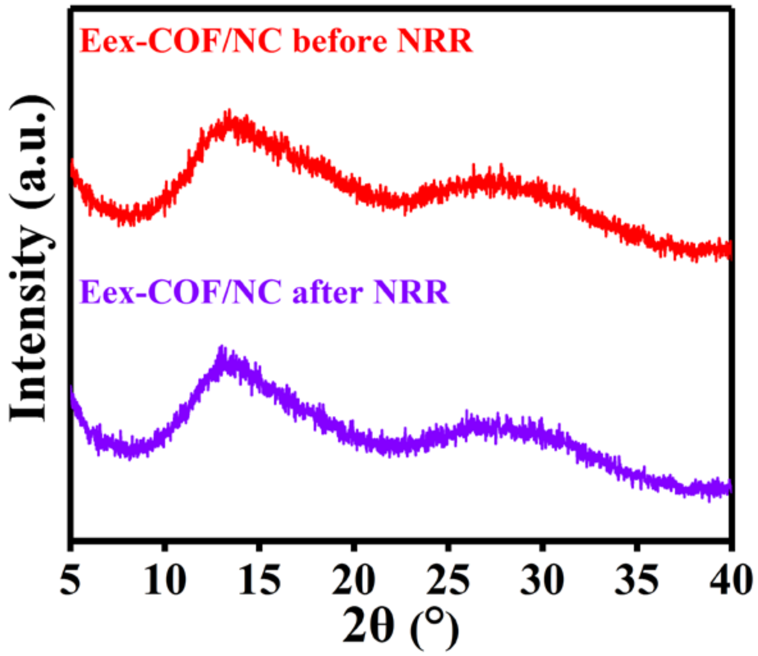


**Supplementary Figure 25.** XRD patterns of Eex-COF/NC before and after NRR electrolysis. This indicates that the Eex-COF/NC maintains amorphic phase through the electrochemical NRR process.

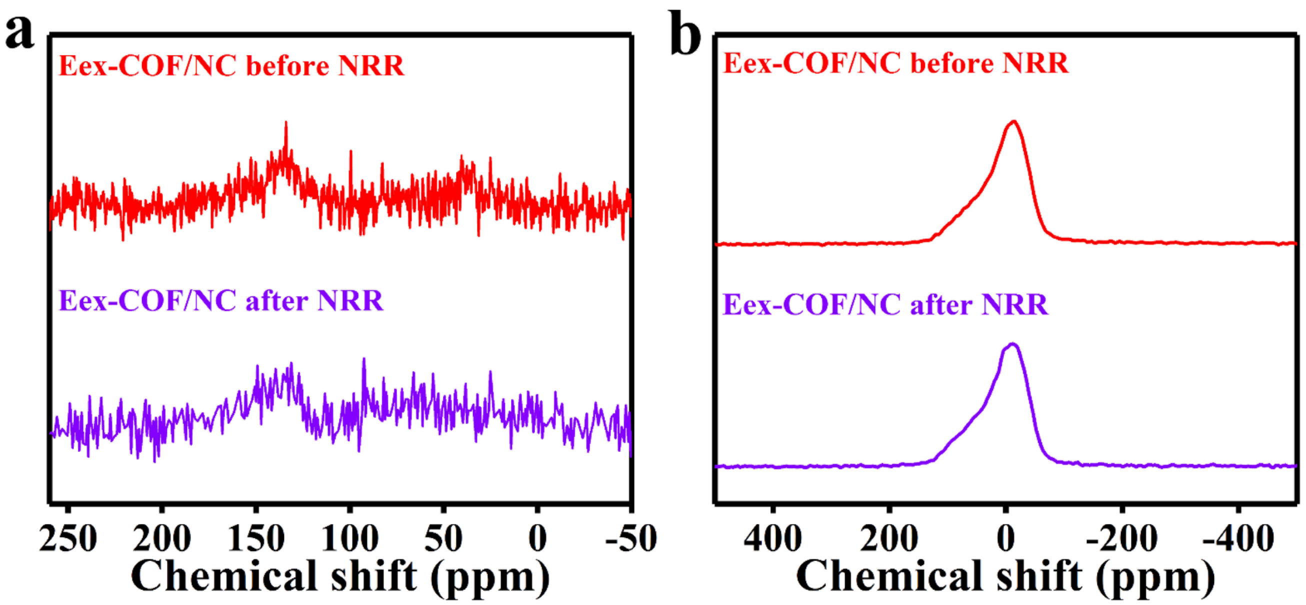


**Supplementary Figure 26. a** $^{13}C$ and **b** $^{11}B$ CP/MAS NMR spectra of Eex-COF/NC before and after NRR electrolysis. This suggests that the chemical environment in Eex-COF/NC keeps steady through the electrochemical NRR process.

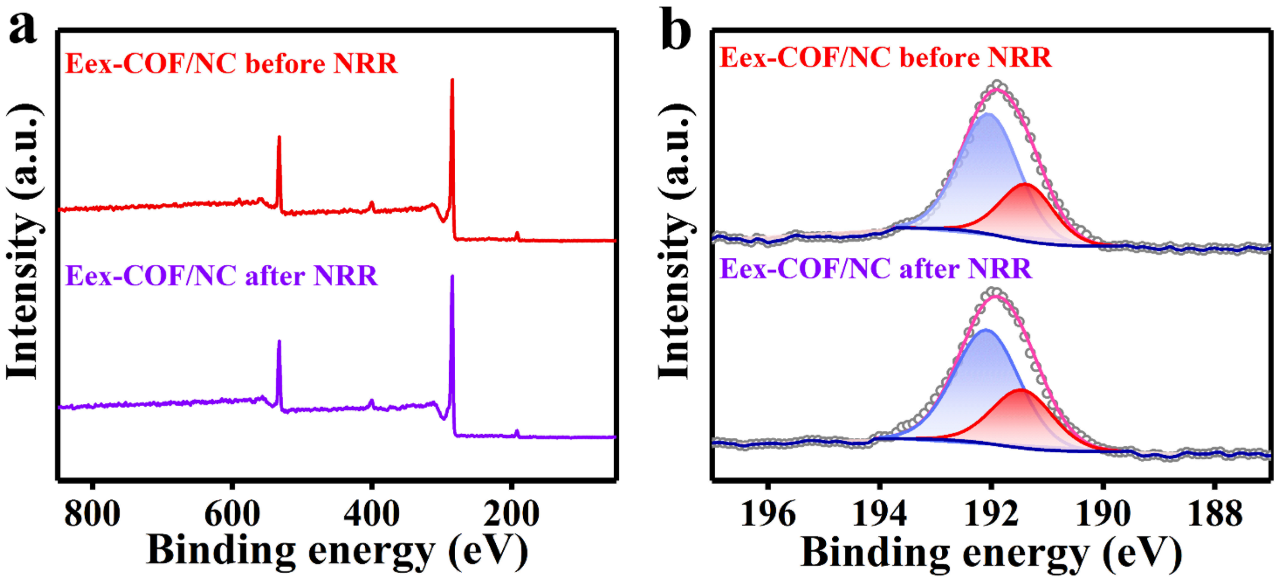


**Supplementary Figure 27. a** XPS survey spectra and **b** high resolution B 1s spectra of Eex-COF/NC before and after NRR electrolysis. The results confirm that the chemical construction of Eex-COF/NC maintains unchanged after NRR test, demonstrating that the excitation state is steady.

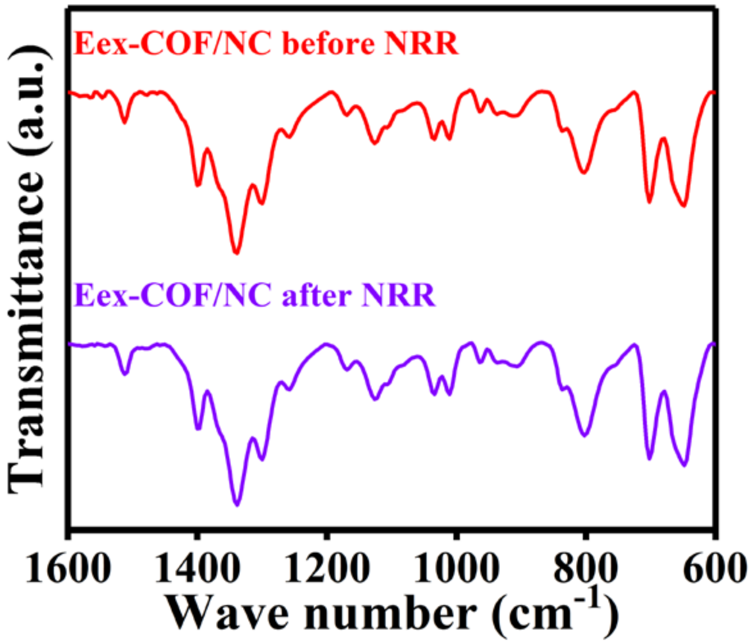


**Supplementary Figure 28.** FTIR spectra of Eex-COF/NC before and after NRR electrolysis. The B-N bonds locating at 815 $cm^{-1}$ and 1370 $cm^{-1}$ remain unchanged after the chronoamperometry measurement.

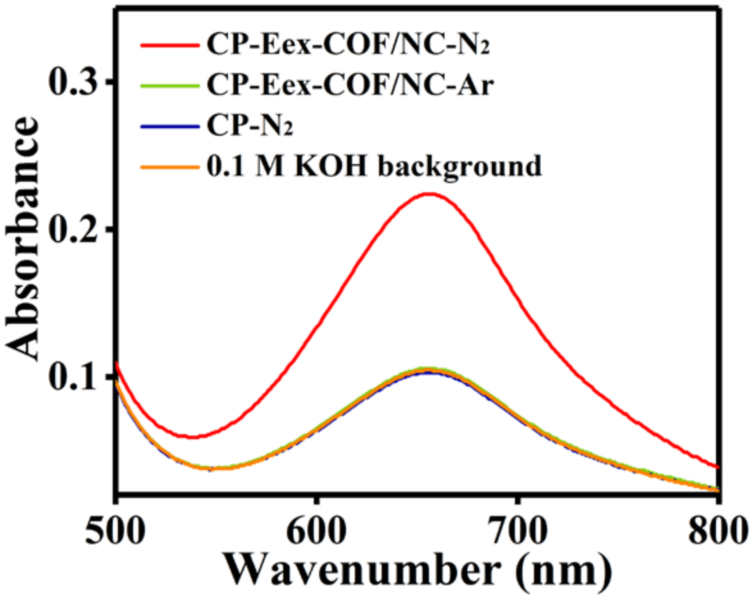


**Supplementary Figure 29.** UV-vis absorption spectra of the electrolytes after electrolysis at –0.2 V vs. RHE under different conditions.

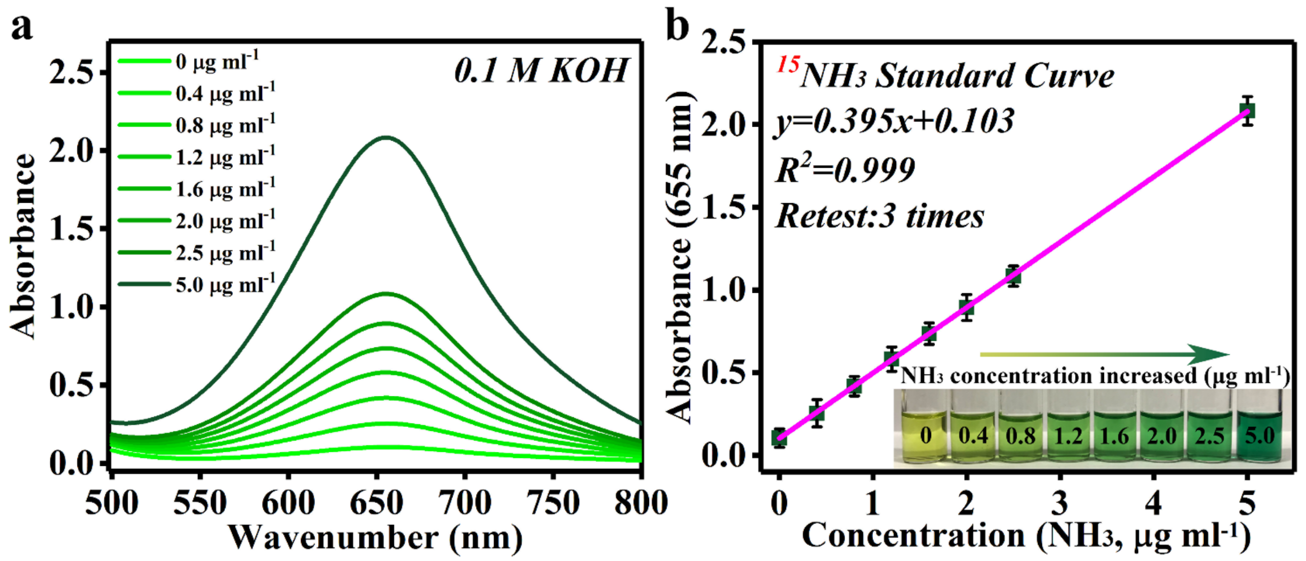


**Supplementary Figure 30. a** The UV-Vis absorption spectra and **b** corresponding calibration curves for the colorimetric $^{15}NH_3$ assay using the indophenol blue method in 0.1 M KOH. The error bars correspond to the standard deviations of measurements over three separately prepared samples under the same conditions.

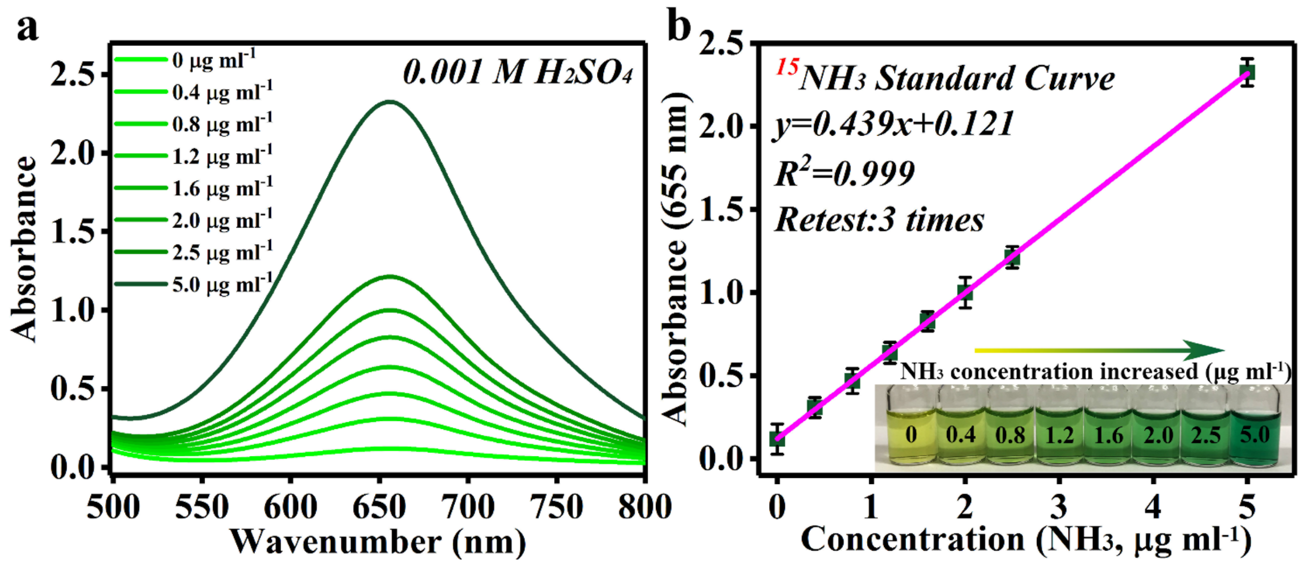


**Supplementary Figure 31. a** The UV-Vis absorption spectra and **b** corresponding calibration curves for the colorimetric $^{15}NH_3$ assay using the indophenol blue method in 0.001 M $H_2SO_4$. The error bars correspond to the standard deviations of measurements over three separately prepared samples under the same conditions.

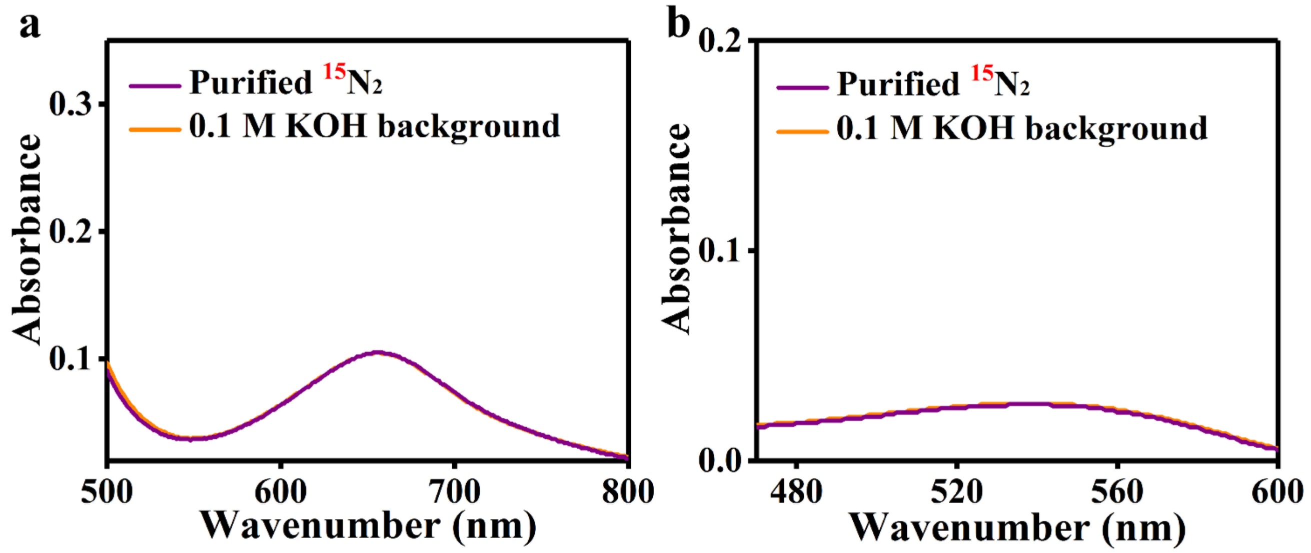


**Supplementary Figure 32.** The UV-Vis absorption spectra of the 0.1 M KOH background and the purified $^{15}N_2$ treated 0.1 M KOH solution using **a** indophenol blue method and **b** N-(-1-naphthyl)-ethylenediamine dihydrochloride spectrophotometric method. The results show that no $NH_3$ or $NO_x$ exists in the purified gas.

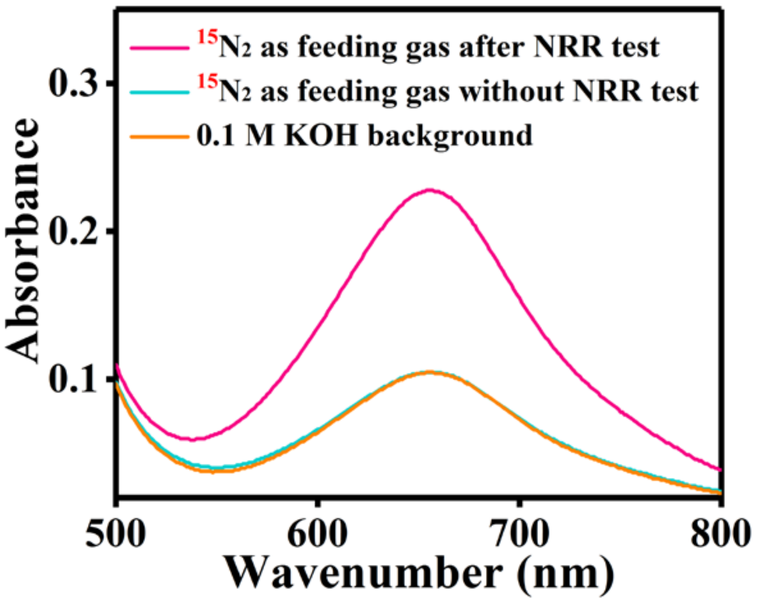


**Supplementary Figure 33.** UV-vis absorption spectra of the electrolytes under different conditions. The pink curve stands for the UV-vis absorption spectrum of the electrolyte after electrolysis using $^{15}N_2$ as feeding gas for both the electrochemical excitation and chronoamperometry measurement.

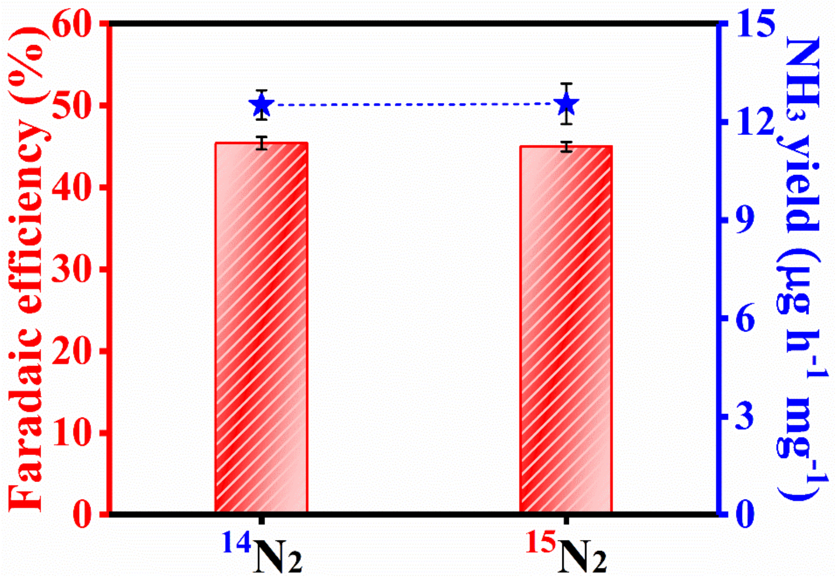


**Supplementary Figure 34.** Comparison of Faradaic efficiency and $NH_3$ yield rate using different feeding gas for NRR at –0.2 V vs. RHE. The error bars correspond to the standard deviations of measurements over three separately prepared samples under the same conditions.

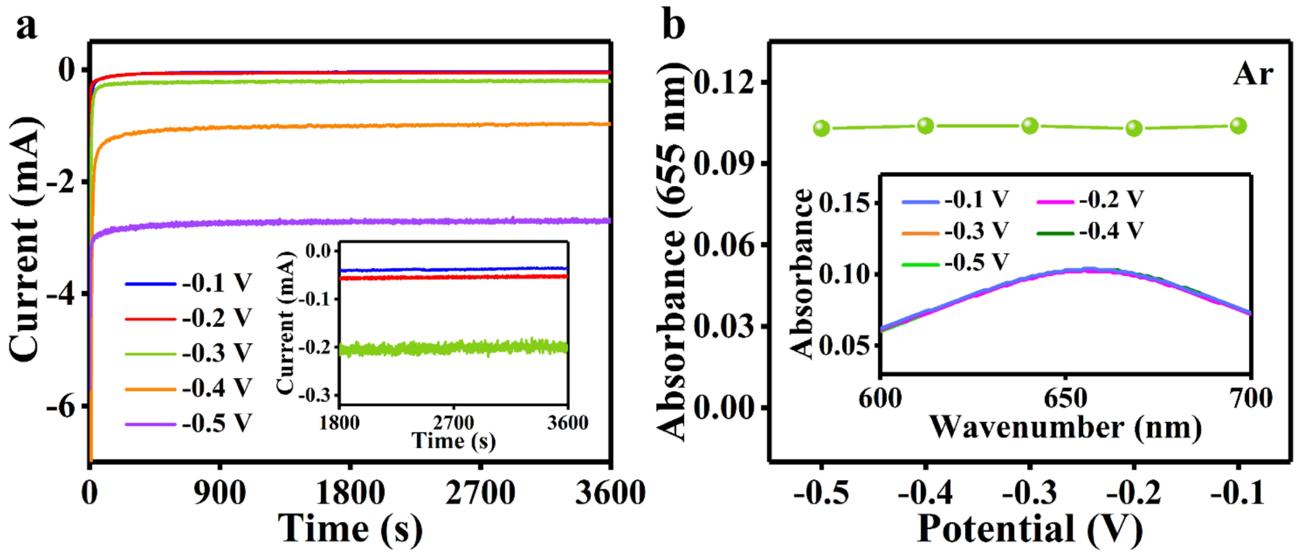


**Supplementary Figure 35. a** Chronoamperometry results of COF/NC under Ar at the corresponding potentials, inset: enlarged view of the curves from –0.3 to –0.1 V vs. RHE. **b** The absorbance value of the electrolyte tested after chronoamperometry measurements at 655 nm under Ar at different given potentials using the indophenol blue method, inset: the UV-Vis spectra.

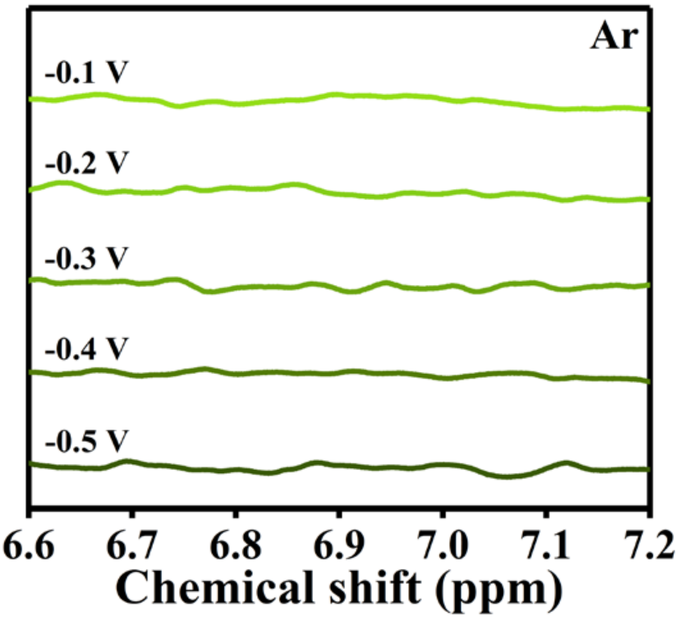


**Supplementary Figure 36.** $^{1}$H NMR spectra of the electrolyte tested after chronoamperometry measurements at different potentials under Ar.

**Supplementary Table 1.** Comparison of the NRR performance of the Eex-COF/NC catalyst with other catalysts reported to date under ambient conditions (room temperature and atmospheric pressure).

| Catalyst | Electrolyte | Potential (V vs. RHE) | Faradaic efficiency (%) | $NH_3$ yield rate | Ref. |
|---|---|---|---|---|---|
| **Pd/C** | 0.1 M PBS | 0.1 | 8.2 | $3.75\times10^{-4}$ μg $s^{-1}$ $cm^{-2}$ | 1 |
| **Au nanorod** | 0.1 M KOH | −0.2 | 3.88 | $4.58\times10^{-4}$ μg $s^{-1}$ $cm^{-2}$ | 2 |
| **Au cluster/$TiO_2$** | 0.1 M HCl | −0.2 | 8.11 | $5.94\times10^{-3}$ μg $s^{-1}$ $cm^{-2}$ | 3 |
| **a-Au/CeO-RGO** | 0.1 M HCl | −0.2 | 10.10 | $2.31\times10^{-3}$ μg $s^{-1}$ $mg^{-1}_{cat.}$ | 4 |
| **$Pd_{0.2}Cu_{0.8}$/rGO** | 0.1 M KOH | 0 | 4.52 | $7.78\times10^{-4}$ μg $s^{-1}$ $mg^{-1}_{cat.}$ | 5 |
| **Amorphous $Bi_4V_2O_{11}$-crystalline $CeO_2$ hybrid** | 0.1 M HCl | −0.2 | 10.16 | $6.45\times10^{-3}$ μg $s^{-1}$ $mg^{-1}_{cat.}$ | 6 |
| **Poly(N-ethyl-benzene-1,2,4,5-tetracarboxylic diimide/C** | 0.5 M $Li_2SO_4$ | −0.5 | 2.85 | $4.39\times10^{-4}$ μg $s^{-1}$ $cm^{-2}$ | 7 |
| **$B_4C$** | 0.1 M HCl | −0.75 | 15.95 | $7.38\times10^{-4}$ μg $s^{-1}$ $cm^{-2}$ | 8 |
| **Few-layer black phosphorus nanosheets** | 0.01 M HCl | −0.6 | 5.07 | $1.16\times10^{-3}$ μg $s^{-1}$ $cm^{-2}$ | 9 |
| **N-doped porous carbon-750** | 0.05 M $H_2SO_4$ | −0.9 | 1.42 | $3.97\times10^{-3}$ μg $s^{-1}$ $cm^{-2}$ | 10 |
| **Polymeric carbon nitride-nitrogen vacancies-4** | 0.1 M HCl | −0.2 | 11.59 | $4.50\times10^{-3}$ μg $s^{-1}$ $cm^{-2}$ | 11 |
| **S-doped carbon nanosphere** | 0.1 M $Na_2SO_4$ | −0.4 | 7.47 | $5.30\times10^{-4}$ μg $s^{-1}$ $cm^{-2}$ | 12 |
| **Nitrogen-doped porous carbon-500** | 0.005 M $H_2SO_4$ | −0.1 | 9.98 | $6.19\times10^{-3}$ μg $s^{-1}$ $cm^{-2}$ | 13 |
| **Eex-COF/NC** | **0.1 M KOH** | **−0.2** | **45.43** | **$3.48\times10^{-3}$ μg $s^{-1}$ $cm^{-2}$** | **This work** |

**Supplementary References**


1. Wang, J. et al. Ambient ammonia synthesis via palladium-catalyzed electrohydrogenation of dinitrogen at low overpotential. *Nat. Commun.* **9,** 1795–1801 (2018).

2. Bao, D. et al. Electrochemical reduction of $N_2$ under ambient conditions for artificial $N_2$ fixation and renewable energy storage using $N_2/NH_3$ Cycle. *Adv. Mater.* **29,** 1604799 (2017).

3. Shi, M. –M. et al. Au sub-nanoclusters on $TiO_2$ toward highly efficient and selective electrocatalyst for $N_2$ conversion to $NH_3$ at ambient conditions. *Adv. Mater.* **29,** 1606550 (2017).

4. Li, S. –J. et al. Amorphizing of Au nanoparticles by $CeO_x$–RGO hybrid support towards highly efficient electrocatalyst for $N_2$ reduction under ambient conditions. *Adv. Mater.* **29,** 1700001 (2017).

5. Shi, M. –M. et al. Anchoring PdCu amorphous nanocluster on graphene for electrochemical reduction of $N_2$ to $NH_3$ under ambient conditions in aqueous solution. *Adv. Energy Mater.* **8,** 1800124 (2018).

6. Lv, C. et al. An amorphous noble-metal-free electrocatalyst that enables nitrogen fixation under ambient conditions. *Angew. Chem. Int. Ed.* **57,** 6073–6076 (2018).

7. Chen, G. –F. et al. Ammonia electrosynthesis with high selectivity under ambient conditions via a $Li^+$ incorporation strategy. *J. Am. Chem. Soc.* **139,** 9771–9774 (2017).

8. Qiu, W. et al. High-performance artificial nitrogen fixation at ambient conditions using a metal-free electrocatalyst. *Nat. Commun.* **9,** 3485 (2018).

9. Zhang, L., Ding, L. –X., Chen, G. –F., Yang, X. & Wang, H. Ammonia synthesis under ambient conditions: selective electroreduction of dinitrogen to ammonia on black phosphorus nanosheets. *Angew. Chem. Int. Ed.* **131,** 2638–2642 (2019).

10. Liu, Y. et al. J. Facile ammonia synthesis from electrocatalytic $N_2$ reduction under ambient conditions on N-doped porous carbon. *ACS Catal.* **8,** 1186−1191 (2018).

11. Lv, C. et al. Defect engineering metal-free polymeric carbon nitride electrocatalyst for effective nitrogen fixation under ambient conditions. *Angew. Chem. Int. Ed.* **57,** 10246–10250 (2018).

12. Xia, L. et al. S-doped carbon nanospheres: an efficient electrocatalyst toward artificial $N_2$ Fixation to $NH_3$. *Small Methods* **2,** 1800251 (2018).

13. Zhao, C. et al. Ambient electrosynthesis of ammonia on a biomass-derived nitrogen-doped porous carbon electrocatalyst: contribution of pyridinic nitrogen. *ACS Energy Lett.* **4,** 377−383 (2019).
